# Shared sequence characteristics identified in non-canonical rearrangements of HSV-1 genomes

**DOI:** 10.1128/jvi.00955-23

**Published:** 2023-11-22

**Authors:** Alina Shitrit, Valerya Nisnevich, Nofar Rozenshtein, Hila Kobo, Hoang Van Phan, Savaş Tay, Moriah Szpara, Matthew D. Weitzman, Nir Drayman, Oren Kobiler

**Affiliations:** 1 Department of Clinical Microbiology and Immunology, School of Medicine, Tel Aviv University, Tel Aviv, Israel; 2 Genomic Research Unit, Faculty of Life Science, Tel Aviv University, Tel Aviv, Israel; 3 Pritzker School of Molecular Engineering, University of Chicago, Chicago, Illinois, USA; 4 Institute for Genomics and Systems Biology, University of Chicago, Chicago, Illinois, USA; 5 Department of Biochemistry and Molecular Biology, Department of Biology, Center for Infectious Disease Dynamics, and the Huck Institutes of the Life Sciences, Pennsylvania State University, University Park, Pennsylvania, USA; 6 Division of Protective Immunity, The Children’s Hospital of Philadelphia, Philadelphia, Pennsylvania, USA; 7 Department of Pathology and Laboratory Medicine, Perelman School of Medicine at the University of Pennsylvania, Philadelphia, Pennsylvania, USA; 8 Department of Molecular Biology and Biochemistry, School of Biological Sciences, University of California, Irvine, California, USA; 9 Center for Virus Research, University of California, Irvine, California, USA; Cornell University Baker Institute for Animal Health, Ithaca, New York, USA

**Keywords:** herpesviruses, DNA rearrangements, microhomology, defective interfering particles

## Abstract

Genomic rearrangements contribute to the enhancement of genetic diversity in populations. However, non-canonical rearrangements (NCRs) such as deletions, insertions, and inversions have the potential to trigger genomic instability. In the case of DNA viruses, NCRs can lead to generation of defective viral genomes (DVGs). To study NCRs in herpes simplex virus type 1 (HSV-1) genomes, we enriched DVGs formation by undiluted serial passaging on various cell types. We found that viral passaging on cell type that enables more viral genomes to initiate replication induces higher amplitude and frequency of cyclic patterns associated with DVGs formation. Despite differences in the rates of DVG accumulation, cell lines displayed comparable quantities of distinct NCRs, indicating that fluctuations caused by DVGs may impose bottlenecks on population genetic diversity. These findings propose additional roles for DVGs in modulating viral genetic diversity. Each cell type exhibited a unique population of NCRs, suggesting that NCRs accumulate in a cell type-specific manner. Interestingly, we identified a higher prevalence of short homologies and short reverse complementary in the parental sequences of NCR junction sites across all cell types. These shared sequence characteristics were also observed in NCRs identified in sequences obtained from clinical samples. The fundamental properties of HSV-1 NCR formation uncovered in this study may have broader implications for other DNA viruses.

**IMPORTANCE:**

Mutations and genetic rearrangements are the primary driving forces of evolution. Viruses provide valuable model systems for investigating these mechanisms due to their rapid evolutionary rates and vast genetic variability. To investigate genetic rearrangements in the double-stranded DNA genome of herpes simplex virus type 1, the viral population was serially passaged in various cell types. The serial passaging led to formation of defective genomes, resulted from cell-specific non-canonical rearrangements (NCRs). Interestingly, we discovered shared sequence characteristics underlying the formation of these NCRs across all cell types. Moreover, most NCRs identified in clinical samples shared these characteristics. Based on our findings, we propose a model elucidating the formation of NCRs during viral replication within the nucleus of eukaryotic cells.

## INTRODUCTION

Viral evolution processes contribute to the emergence of new viral variants, drug-resistant strains, and zoonotic infections. The rapid dynamics of viral evolution allows selection of genetic differences both *in vitro* and *in vivo* within hosts and across populations [as was observed during the severe acute respiratory syndrome–related coronavirus 2 (SARS-CoV-2) pandemic (1, 2)]. The major forces driving viral evolution are high mutation rates, high recombination rates, and large population sizes. DNA viruses have lower mutation rates than RNA viruses, and thus, the role of recombination for these viruses seems to be more dominant as a way of generating genetic diversity (3).

A common genetic variation observed among many virus populations is the accumulation of incomplete genomes, also known as defective viral genomes (DVGs) or defective interfering particles (4). DVGs are defined as genomes that are unable to complete a replication cycle without the complementing functions of co-infecting complete viral genomes. Following passaging of RNA and DNA viruses *in vitro* at high multiplicity of infection (MOI), a cyclic pattern between infectious particles and DVGs is observed (5–8), resulting in bottlenecks in the infectious particles population when DVGs accumulate. In RNA viruses, DVGs have also been detected *in vivo* (9–12). For influenza virus, DVGs found *in vivo* were similar in sequence to the DVGs found previously *in vitro* (10). Several functions have been suggested for these defective genomes during infection, including immune modulation and establishment of RNA virus persistence (4, 13). DVGs compete for resources with the intact viral genomes and are thus being explored as potential antiviral treatments and vaccines (14–16).

Herpes simplex virus type 1 (HSV-1) belongs to the alphaherpesviruses subfamily and is a large, double-stranded DNA virus that infects more than half of the human population and is associated with significant morbidity. Following infection, most individuals will maintain latent viral genomes for the rest of their lives, and these persistent genomes periodically reactivate. In most cases, symptomatic reactivation leads to the formation of herpetic lesions (cold sores), which are thought to be the most contagious periods due to the high titer of viruses found in these lesions (17). As HSV-1 infection is rapid and lesions last for 7 to 10 days, it is likely that within the lesions several rounds of high multiplicity infections can occur.

Sequencing of clinical HSV-1 isolates has suggested that intergenomic recombination is the major source of genetic diversification of these viruses (18). Recombination events have also been observed between HSV-1 and the distantly related virus HSV-2 (19).

Like all herpesviruses, HSV-1 genomes replicate within the nucleus of the infected host cell at specific domains known as replication compartments (20). Viral and host proteins required for viral genome replication accumulate at these replication compartments. In most cases, each replication compartment originates from a single incoming genome, and only a limited number of incoming viral genomes initiate expression and replication per cell (21, 22). The viral replication compartments grow and coalesce during infection, providing the opportunity for intergenomic recombination (23, 24).

**Fig 1:**
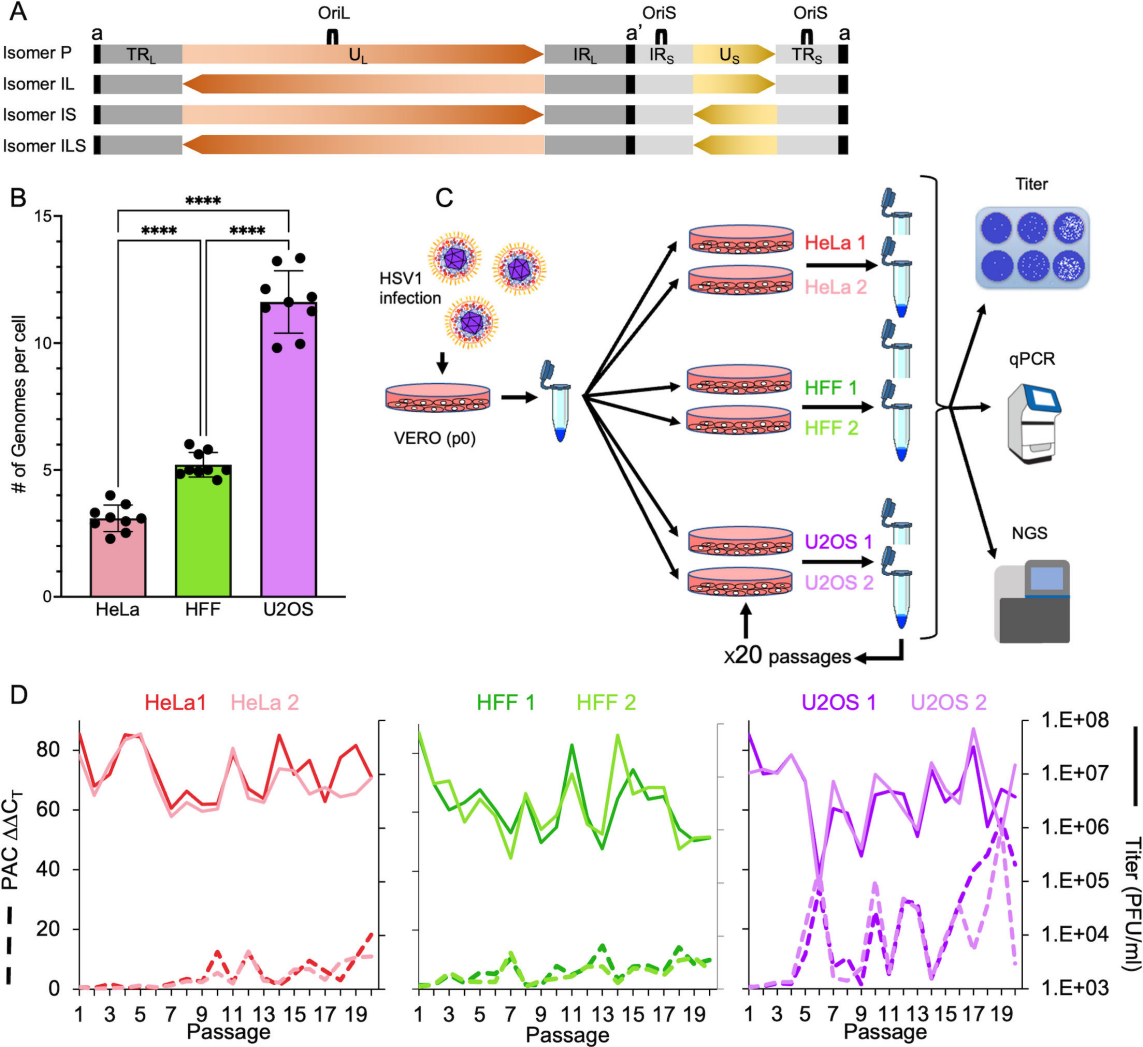
HSV-1 infectivity cyclic patterns vary among cell lines. (**A**) A schematic illustration of the HSV-1 isomers. The viral unique long and short (U_L_ orange and U_S_ yellow, respectively) regions are shown with arrowheads to indicate directionality. Terminal and internal repeat regions (TR and IR respectively), are marked in shades of gray and the a’ sequence is marked in black. Sites of long and short origins of replications (OriL and OriS, respectively) are also marked as illustrated (not to scale). (**B**) The number of viral genomes that are expressed per cell varies among different cell lines. HeLa (red), human foreskin fibroblast (HFF; green), and U2OS (purple) were infected at MOI 20 with an even mixture of the three fluorophore-expressing HSV-1 recombinants. Images taken 6–8 hours post infection (HPI) were used for calculating the average number of genomes expressed per cell. Each bar represents three biological repeats, in each three images were analyzed, represented as dots. Error bars represent standard deviations between images. ****P < 0.0001; by one-way analysis of variance. (**C**) A schematic illustration of experimental protocol for 20 undiluted passaging of HSV-1 on different cell lines. A plaque purified stock of HSV-1 was grown on Vero cell to initiate the infection process (**p0**). Two different 10 cm plates for each cell line (HeLa, HFF, or U2OS) were infected from the original p0 stock. At 48 HPI, cells and medium were collected, and 2 mL of the sample was used to infect the next round of infection. The remainder of the sample was used for further analysis: titer was analyzed by plaque assay, quantitative PCR (qPCR), and sequencing. This process was repeated for 20 times. (**D**) The titer (full lines) and packaging signal (PAC) copy numbers compared to UL11 gene (dashed lines) at the different passages are shown for each replicate in each cell line as indicated. Color coded as indicated at the top where darker color represents replicate 1 and the brighter for replicate 2. We have chosen the colors from colorblind safe color palette using the Chroma.js Color Palette Helper (https://gka.github.io/palettes/).

HSV-1 genome consists of two distinct regions referred to as long (L) and short (S). Each region has a unique sequence and flanking inverted repeat sequences (Fig. 1A). There are three copies of the ~400 bp–500 bp a’ sequence found at the edges of the genome and at the L-S junction, and these contain the packaging signal (25). The a’ sequence provides a possible mechanism for the formation of the four isomers of the genome (26, 27) (Fig. 1A). The viral genome possesses three origins of replications: one at the U_L_ region known as OriL and two in the short repeat regions R_S_ known as OriS.

HSV-1 encodes seven proteins that form the viral replication machinery and are essential for viral DNA synthesis (28). In addition, the virus harnesses host proteins required for efficient replication. Viral recombination is tightly associated with replication, and has been suggested to similarly depend on both viral and host proteins (29). The homology-based recombination observed during HSV-1 replication is proposed to be mediated via the single-strand annealing mechanism (30). Non-homologous recombination events are also observed during HSV-1 genome replication, resulting in non-canonical rearrangements (NCRs) such as deletions, duplications, and inversions (27). HSV-1 DVGs contain large deletions in the viral genomes indicating they result from NCR events; however, NCR events could result in infectious genomes (non-DVG).

Two classes of HSV-1 DVGs were identified decades ago and were proposed to arise through rearrangements producing tandem repeats of truncated short sequences from the viral genomes (5, 27, 31, 32). Both classes include the packaging signal (PAC) and differ in the origin of replication, class I containing the OriS sequence and class II containing the OriL sequence (25, 33). However, the exact boundaries of the genetic material in the DVGs were not characterized.

Here, we examined at unprecedented resolution the genomic rearrangements that occur during HSV-1 replication. By serially passaging HSV-1 in different cell lines, we shed new light on the cell type-specific and shared patterns of DVGs formation and the sequence properties that underlie these viral NCRs. These sequence properties were enriched even further from sequences obtained from uncultured clinical samples. Thus, our findings indicate the role of these sequence properties in NCR formation *in vivo* and suggest that similar DVGs may be observed both *in vivo* and *in vitro*. Our suggested models provide new insight to the mechanisms involved in viral replication and recombination processes.

## RESULTS

### The cyclic patterns of viral titers vary among cell lines in accordance with the number of genomes initiating replication

Previous work from our lab showed that the number of viral genomes initiating replication and expression is cell type dependent (34, 35). We hypothesized that cells which allow more incoming genomes to initiate expression and replication (22) will display increased number of point mutations and non-canonical rearrangements including DVGs. Based on our previous studies (34, 35), we selected three human cell lines that have a wide variability in the number of genomes that enter their nuclei and start replication: HeLa (epithelial cell from adenocarcinoma), immortalized HFF (human foreskin fibroblast), and U2OS (moderately differentiated osteosarcoma cells with epithelial morphology). At MOI 20, U2OS cells support the highest number of genomes initiating expression (11.6 genomes), followed by HFF and HeLa with averages of 5.2 and 3.1 genomes, respectively (Fig. 1B).

To investigate the genetic diversity and DVG formation during viral replication at high MOI conditions, we began our study from a plaque purified stock raised on Vero cells, which was defined as passage zero (p0). We then passaged the virus 20 times, without dilution, in the three cell lines, in two replicates, and then analyzed the titers and genetic variability of the viral populations through plaque assays, quantitative PCR (qPCR) and next-generation sequencing (NGS; see Materials and Methods and Fig. 1C). Viral titers showed a cyclic pattern in all cell types throughout the experiment (Fig. 1D, full lines), suggesting the formation of DVGs (5, 36). Interestingly, fluctuations in U2OS viral titers were of higher amplitude and frequency compared to HFF. The titer changes in HeLa had the lowest amplitude and frequency.

### Defective viral genomes vary among cell lines in accordance to the number of genomes that initiate replication

To verify the extent to which these fluctuations in viral titers are due to accumulation of DVGs, we measured the copy number of two elements that are known to be found in HSV-1 DVGs: the origin of replication (OriS/OriL) and the packaging signal (PAC) (25, 37). We observed fluctuations in PAC copy numbers as we progressed through the 20 passages (Fig. 1D, dashed lines). The amplitude and frequency of the fluctuations in PAC abundance are highest in U2OS and lowest in HeLa cells, mirroring the fluctuations in viral titers [*r* = −0.23, *P*-value = 0.012, CI= (−0.05, –0.39), *n* = 120, Pearson’s product-moment correlation, for all cell line replicates together).

Similarly, a negative correlation between OriS abundance and the titer was also observed [*r* = −0.29, *P*-value = 0.012, CI = (–0.12, –0.45), *n* = 120, Pearson’s product-moment correlation, for all cell line replicates together Fig. S1]. However, OriL abundance did not change throughout the 20 passages and, thus, no correlation to the titer was observed (Fig. S1). These results indicate that in our experiments, most HSV-1 DVGs are of class I, containing OriS and very little, if any, are of class II DVGs. Taken together, our findings suggest that cell lines which allow higher number of viral genomes to initiate replication per cell can influence the fluctuations in DVGs formation (i.e., the higher number of genomes starting replication, the higher amplitude and frequency of the fluctuations).

DVGs are lacking crucial parts of the viral genome and are therefore inherently dependent on co-infection (38). Under low MOI, the probability of co-infections decreases, and thus the potential for replication and packaging of DVGs. To demonstrate further that the titer fluctuations are due to DVG formation, we conducted passage 21 at MOI 0.01, which should result in elimination of most of the DVGs. As expected, in passage 21, the OriS copy numbers were similar to the p0 levels for all cell lines (Fig. S2A, dashed lines), supporting the hypothesis that these OriS and PAC are prevalent in DVGs and their copy numbers provide an estimate for the DVGs percentage in each passage.

### Sequencing of viral passaging shows limited variation in single nucleotide polymorphism

To further characterize the differences between DVGs formation in the different cell lines, we monitored genomic sequence changes along the series of undiluted passaging. We used NGS to sequence the original stock (p0) and all six samples (three cell lines in duplicates) for 14 of the passages (out of the 21 passages; 1, 4, 7, 10, and 12–21). Samples were sequenced to an average depth of 6.7 million 250 bp paired-end reads (see Table S2 for NGS statistics). Our sequencing data are in good overall concordance with the qPCR measurements, comparing OriS sequencing reads to qPCR copy numbers [cor = 0.616, *P*-value = 1.9e − 09, CI = (0.45, 0.73), *n* = 78, Pearson’s product-moment correlation, Fig. S2A and B].

**Fig 2:**
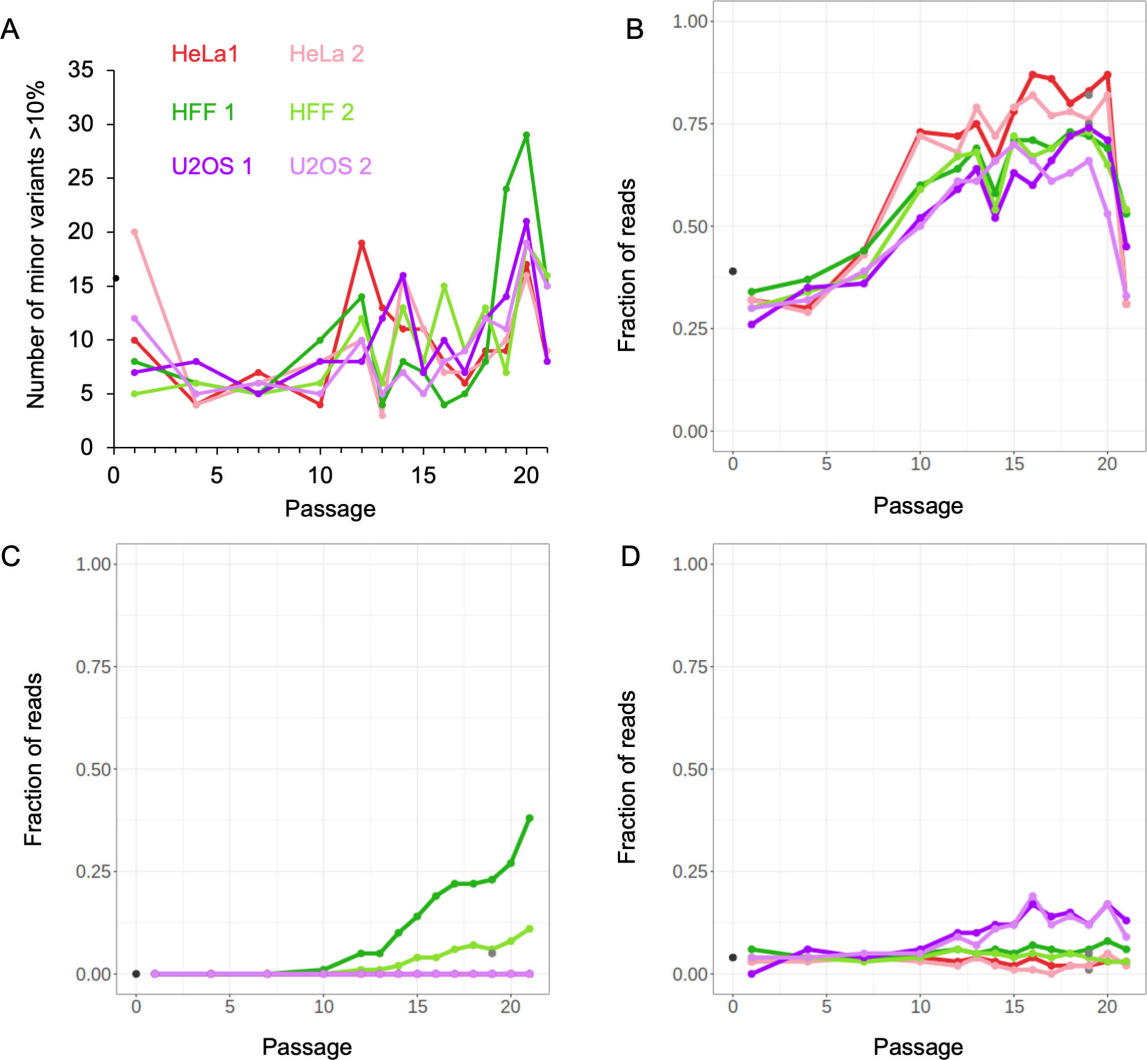
Minimal differences in single nucleotide polymorphism during HSV-1 undiluted passaging among the cell lines. (**A**) The number of different minor variants detected in each passage for each cell type replicate: p0 (black), HeLa (shades of red), HFF (shades of green), U2OS (shades of purple), darker color represents replicate 1 and the brighter for replicate 2 as indicated. Minor variants were counted only if the reads reached more than 10%. (**B–D**) Examples of ubiquitous in US1 gene (**B**) or cell specific in gD and gE genes (**C and D**) minor variants emergence (as percent of total reads) during the passaging on the different cell lines, color coded as (**A**). The specific nucleotides are (**B**) 134215C to G, (**C**) 140635G to T, and (**D**) 143508C to T.

We analyzed the viral sequences to reveal the emergence and dynamics of both single nucleotide changes (minor variants) and NCRs during passaging. We first focused on quantitating accumulation of minor variants. Using the AccuNGS tool (39) with the recommended accurate cutoff of 10%, we detected only 124 unique minor variants in our data set (both synonymous and non-synonymous and in non-coding regions). Of these, 15 were already found above 10% in p0. We observed a slight increase in minor variants in passages 18–20 and a decrease in passage 21 (Fig. 2A), indicating that some of these minor variants might be in non-infectious genomes (40). We did not observe a significant difference in the overall amount of minor variants between cell types (Fig. 2A). Only one minor variant prevalence increased during passaging in all cell types. This point mutation in the US1 gene was already present in 40% of the viral sequences at p0, and increased to ~85% of sequences at passage 20 and decreased back to ~40% at passage 21 (Fig. 2B). This minor variant occurs 39 nucleotides upstream of the start of ICP22 (US1), changing a C to G in a pattern of (GGGCGGGGGG). Two examples for cell line-specific minor variants are shown (Fig. 2C and D). In HFF, a minor variant appeared at passage 10, and its prevalence increased during passaging, and then further increased in passage 21. This minor variant was positioned in the gD protein, and results in a change of the amidic glutamine to a basic histidine. Another cell type-specific minor variant was found during passaging in the U2OS cell line. This minor variant was present at p0, was detected as increasing at passage 12, and slightly declined by passage 21. This minor variant is positioned within the gene for the gE protein where it changes a positively charged arginine to cysteine. Overall, our sequencing data revealed that the amount and prevalence of minor variants during passaging is limited and is not associated with DVGs formation.

### Non-canonical rearrangements accumulate in a cell type-dependent manner

**Fig 3:**
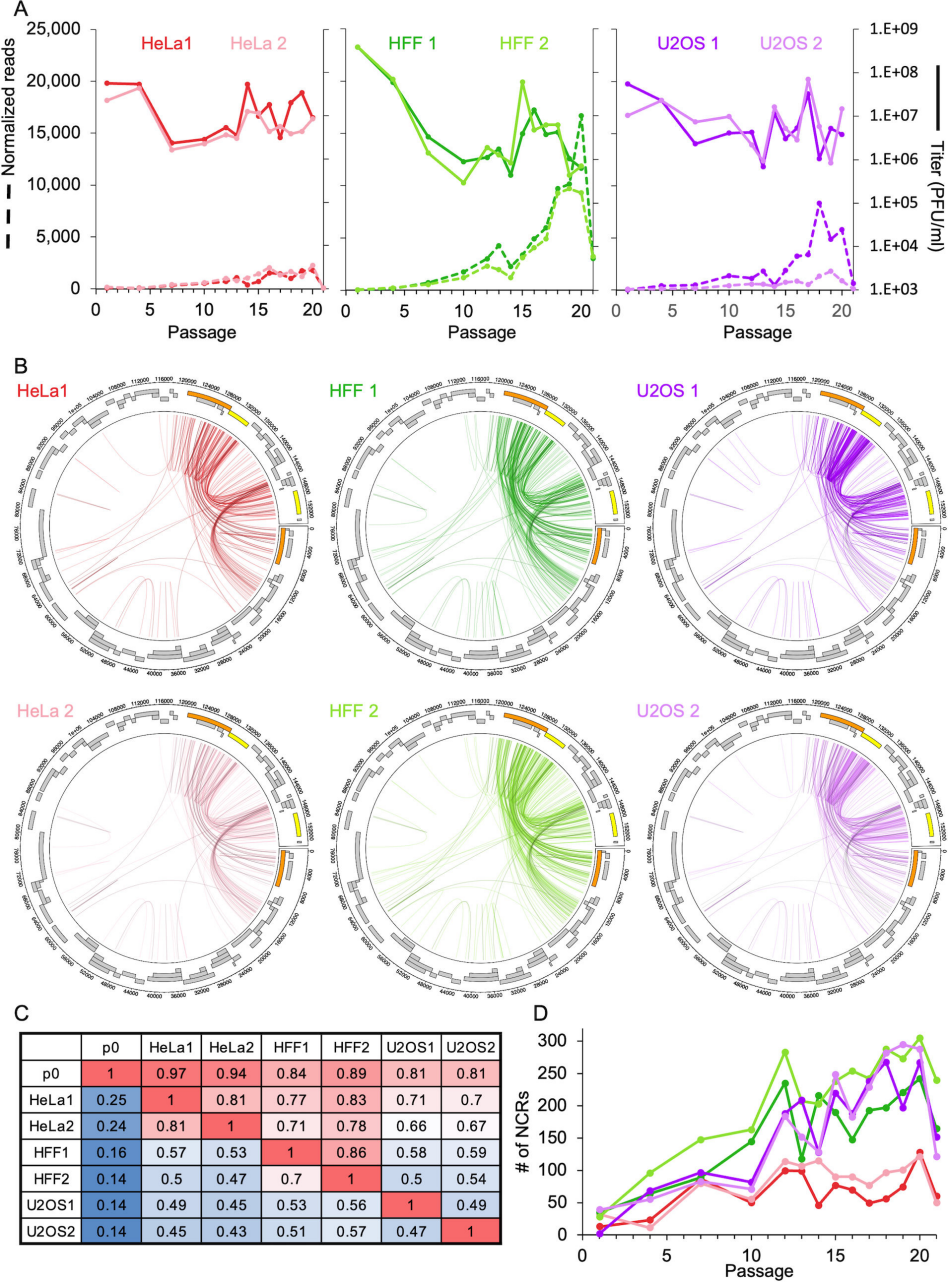
NCRs vary among different cell lines during HSV-1 undiluted passaging. (**A**) The titer (full lines) and the summed number of normalized reads supporting NCRs (dashed lines) at the different passages are shown for each replicate in the specific cell line indicated (color coded as Fig. 1D). (**B**) Circus genome plots of NCR junctions throughout the viral sequence (assembled p0 genome) presented for all samples (two replicates for each cell line as indicated, color coded as above, and at each circle, NCRs detected at the p0 presented on in black top of each color). Each unique NCR is plotted as one line from the start to the end positions. Viral genes are marked in gray boxes, and the LAT gene and RS1 are labeled in orange and yellow, respectively, to denote the repeat region of the genome. The graph represents all unique NCRs over all the passages. (**C**) Similarity of the unique NCRs among the replicates of each cell line and compared with p0. Similarities were calculated by comparing all the individual NCRs in all the passages of each cell line replicate (as indicated by the row name) to the individual NCRs in other cell line replicates (as indicated by the column name). The sum of all matched-pair NCRs was divided by total NCRs of each replicate (as indicated by the row name). (**D**) Number of new NCRs detected per cell line sample along the passages. In each passage, the number of individual NCRs that are not detected in p0 are plotted. Color coded as (**A) and (B**).

To analyze NCR events such as insertions, deletions, and inversions, we extracted reads that did not align to either the human genome or the p0 reference genome. We quantified the number of NCRs through passaging via the ViReMa tool [ViReMa (41) post-processing in Materials and Methods]. We observed a negative correlation between the amount of NCRs and the viral titer [*r* = −0.42, *P*-value = 0.00011, CI = (–0.22, –0.59), *n* = 78, Pearson’s product-moment correlation, for all cell line replicates together, Fig. 3A]. This suggests that most of the DVGs arise through NCRs.

Over the entire 20 passages, we detected hundreds of different NCRs (an average of 242, 363, and 363.5 different NCRs in HeLa, HFF, and U2OS cells, respectively) compared to 63 NCRs at p0 (Fig. S3A; Table S4). While hundreds of NCRs were detected, most reads came from a small number of highly frequent NCRs (the top 10 most frequent NCRs accounted for 70%–85% of the NCRs supporting reads in the different samples, except for U2OS2, in which they accounted for 45%). The distribution of NCRs distances (according to canonical P isomer) shows that most NCRs are either longer than 100,000 bp or shorter than 40,000 bp (Fig. S3B). Interestingly, different patterns of distances are observed among the different cell lines; however, similar patterns are observed between cell line replicates. Specifically, a third of the NCRs of U2OS replicates are between 1,000 bp and 5,000 bp.

To visualize the distribution of NCRs in the different cell lines, we plotted the total different NCRs from start to end along the circular genome (Fig. 3B). Most NCRs in all cell lines had at least one end around or within the repeat regions. Although there are similarities between the circular plots among the different cell line replicates, a specific pattern for each cell line can also be observed. For example, U2OS replicates have many NCRs that start and end within the short repeat region (Fig. 3B, left panels) supporting the formation of more DVGs [tandem repeats of the OriS and PAC as previously observed (25)] in this cell line.

Since each NCR junction might be present in each of the four different isomers of the viral genome (Fig. 1A), we present all possibilities in coverage plots (Fig. S4). The NCR coverage plots represent the frequency of each NCR start and end position for each sample. Interestingly, cell line replicates that were passaged separately produce very similar NCR coverage plots, while the profile of NCRs changed dramatically between different cell types. HeLa replicates maintain the p0 coverage plots distribution with higher supporting reads suggesting accumulation of existing NCRs (Fig. S4 and S5).

In all cell line replicates, the NCRs start to accumulate at very early passages (around passage 7). The coverage plot distributions are similar, while the number of supporting reads fluctuates throughout the passages (Fig. S5). As observed in Fig. 3A and Fig. S2A and B, the total number of normalized supporting reads per passage correlates with DVG accumulation, and negatively correlates with the infectivity. Taken together, these results indicate that NCRs forming DVGs start to accumulate at early passages in a non-random fashion and in a cell type-dependent manner.

### Non-canonical rearrangements diversification is cell dependent

To quantify the similarities among the total different NCRs (at all passages) within each cell line replicate to all other replicates, we showed that all the cell lines accumulated new NCRs that were not observed in p0 (Fig. 3C, column 1). Figure 3C suggests that passaging on U2OS cells may generate the most diversified NCR populations in comparison to those from HFF and HeLa cells.

We plotted the number of newly observed different NCRs (not detected in p0) along the passages for each replicate (Fig. 3D). From passage 12, replicates from HFF and U2OS cells increase in the number of new NCRs compared to HeLa. These new NCRs are not very frequent and are supported by a low number of normalized reads, and thus are not visible in the coverage plots (Fig. S5).

### Short homology sequences are frequently found in NCRs

**Fig 4:**
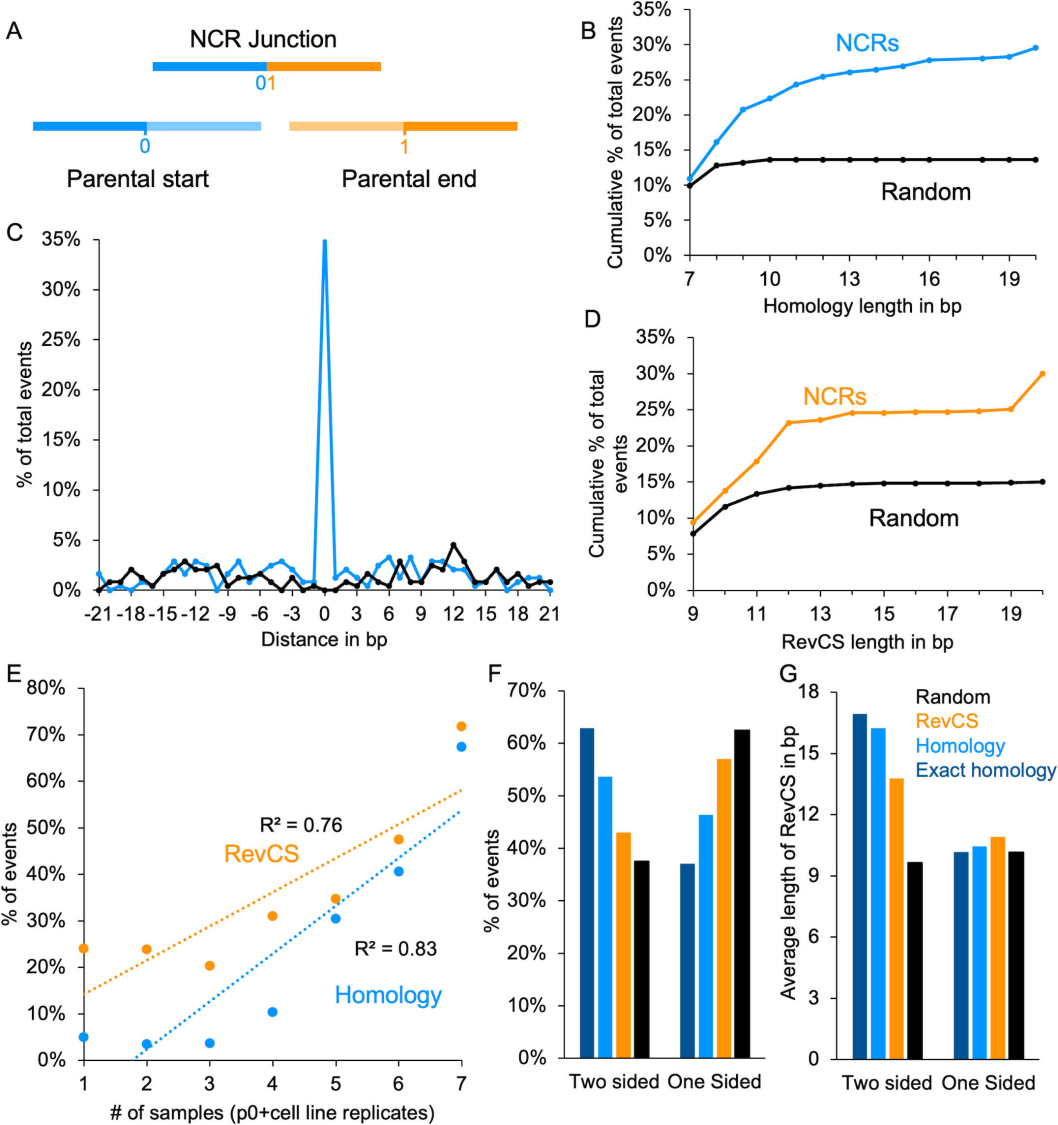
Homology sequences and reverse complement sequences are present near NCR junction sites. (**A**) Illustration of NCR junction site. Two parental genomes (start and end) are present until the 0 (start) and from the 1 (end) positions, thus creating the new genome junction. (**B**) Percentage of NCR events in which homology between the parental genomes was detected accumulating over different length (bp). Results from all NCR events detected in this study (light blue) are compared with random start and end sites within the herpes genome (black). (**C**) Distribution of maximal distance in base pairs of the microhomology from the junction site either at the start (negative numbers) or the end (positive numbers) parental sequences. In cases in which equal distance was observed in both parental sequences, the distances were randomly distributed between the positive and negative numbers. Color coded as (**B**). (**D**) Percentage of NCR events that have reverse complement sequences (RevCS) near the junction sites of the NCRs in different length (in bp). Results from total events detected in this study (orange) compared with random start and end sites within the herpes genome (black). (**E**) Percentage of RevCS (orange) or NCR events with exact site homology sequences (light blue) out of total NCR events distributed over the number of cell line replicates in which the events (homology or RevCS) were detected. *R*^2^ for each correlation is presented. (**F**) Percentage of all NCRs with RevCS are distributed according to the presence of the RevCS at either one parental side of the NCR junction site (one-sided) or between the two parental genomes (two-sided). Random start and end (black), NCRs with RevCS (orange), NCRs with RevCS and homology sequences (light blue), and NCRs with RevCS and exact site homology sequences (blue). (**G**) Average length of RevCS at either side of the NCR junction site (one-sided) or between the two parental genomes (two-sided). Color coded as (**F**).

We explored the possibility that sequence preferences contribute to NCRs formation. We first checked whether any sequence motifs were shared among NCRs sites (in proximity of 25 bp from the junction site), but did not find any. Next, we searched for short homology sequences in proximity to the junction start and end sites, since microhomologies can often be observed between parental sequences that originate from rearranged junctions (42) (Fig. 4A). Out of the 806 unique NCRs detected (in all cell lines), 243 (30%) contained homology sequences of 7 bp–20 bp. To assess the significance of this finding, we randomly selected 970 start and end positions from the HSV-1 genome and analyzed them for the presence of microhomologies. Only 132 (13.6%) showed homology sequences of 7 bp–10 bp (and none showed homology >10 bp). Comparing the distribution of homologous length between the NCRs identified and those randomly generated, we observed significantly longer homology sequences in those from detected NCRs (Fig. 4B, exact two-sample Kolmogorov-Smirnov test, two-sided *P*-value = 0.0025).

Microhomologies can be observed either at or near the exact junction site (43). To evaluate the distribution around the exact junction site, we plotted the maximal distance in base pairs from the start parental sequence (<0) or from the end parental sequence (>0) (Fig. 4C). In a fraction of the NCRs (89 events or 37%), we observed short homology at the exact site, compared to the random where none were found (*P*-value = 4.44e − 16, two-sample independent proportion test).

We divided all our identified NCRs into seven categories based on their prevalence in the seven different cell line replicates [p0 (Vero), HeLa1 and 2, HFF1 and 2, and U2OS1 and 2], i.e., the category number represents the number of cell line replicates in which that specific NCR was identified. NCRs that were independently detected in multiple cell line replicates had, on average, longer homologies and had significantly more exact junction site homologies (Fig. S7A and B, respectively). Taken together, our results suggest that short homology (7 bp–20 bp) is likely a common feature in HSV-1 NCRs.

### Reverse complement sequences are frequently found in NCRs

One of our identified NCRs with microhomology showed deletion of the OriL sequence (Fig. 6A). The OriL sequence has been suggested to form a hairpin-like secondary structure (33). Therefore, we searched the NCRs start and end proximity for reverse complement sequences (RevCS) that may form secondary DNA structures. We allowed for the reverse complement in either one parental side or between both parental sequences. We identified 242 NCRs (30%) with RevCS longer than 9 bp, while in the search of randomly generated junctions, only 144 (15%) were detected (Fig. 4D). RevCS were significantly longer in NCRs sequences than in the random data set (Fig. 4D, exact two-sample Kolmogorov-Smirnov test, two-sided *P*-value = 1.5e − 14).

We compared the number of events (RevCS or microhomology found at the exact junction site) to the total NCRs in each of the seven cell line prevalence categories. We observed a correlation between the NCRs that were prevalent in more cell lines to the likelihood that RevCS or exact site microhomology sequences were identified (Fig. 4E). Similar to short homology findings, longer RevCS (above 12) are more common in more cell line replicates (Fig. S6C).

Our results suggest that both short homology and RevCS contribute to HSV-1 NCRs formation. We found that NCRs with RevCS had a higher probability to have short homology, 110/242 (45%), compared to NCRs without RevCS in which only 133/564 (24%) had short homologies (*P*-value = 3.25e − 11, two-sample independent proportion test). To explore this dependency between the short homology and RevCS, we characterized the distribution of short homology and exact junction site homologies in RevCS present in one parental side or RevCS from both parental sequences (Fig. 4F). More RevCS were identified at one parental side similar to random start and end junction positions. The NCRs with RevCS and homology or exact site homology sequences are more enriched for two sides compared to all RevCS (exact McNemar test, OR = 1.47 and 2.95, *P*-value = 0.025 and 6.7e − 10, respectively; Fig. 4F). In addition, the RevCS lengths were found to be longer on average for two sides of the parental sequences (Fig. 4G). Thus, NCRs with RevCS at one side or between the two sides may have different properties.

### Data from clinical isolates support sequence drivers of NCRs formation

**Fig 5:**
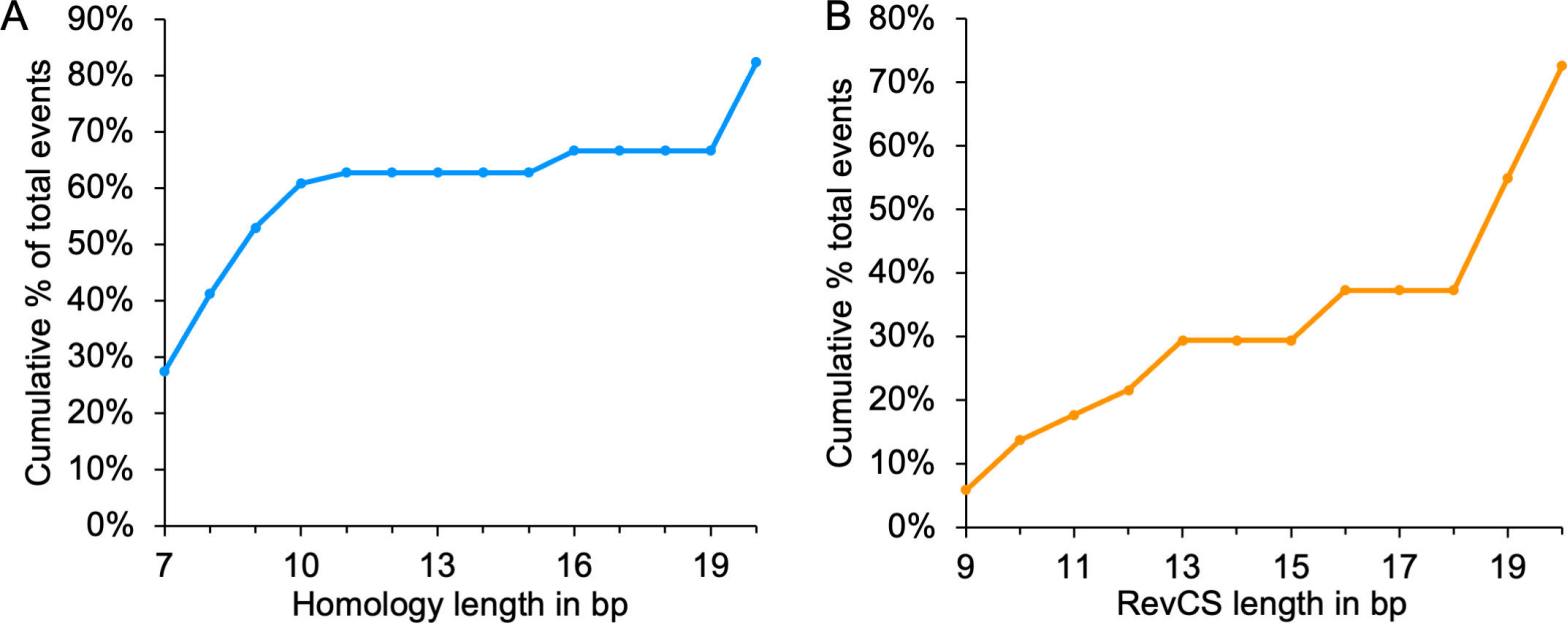
Homology sequences and reverse complement sequences are more prevalent near NCRs from clinical samples. (**A**) Percentage of NCR events in which homology between the parental genomes was detected accumulating over different length (in bp). (**B**) Percentage of NCR events that have RevCS near the junction sites of the NCRs in different length (in bp) form total events detected in the four clinical samples tested.

We identified sequence properties that are common in the HSV-1 NCRs detected during the undiluted passaging in different cell lines. We wished to explore whether these NCRs and properties can be found also in sequencing from clinical isolates. We retrieved NGS data of four HSV-1 clinical isolates from previous studies, including a transmission pair of mother to baby [mother blood, newborn blood, and skin (44)], and an additional unrelated genital sample (45). We identified 51 NCRs (after clustering) in the clinical samples, of which 42 (82%) possessed microhomologies (7 bp–20 bp) (Fig. 5A), out of which, 62% are on the exact junction site position. Most of the NCRs, 37 out of the 51 (72%), were found with RevCS (9 bp–14 bp) (Fig. 5B). In both cases, the graphs suggest that many of the homologies and RevCS are even longer than the limit of our search. Thirty-two out of the 37 (86.4%) that were found with RevCS also had short homologies (7–20).

**Fig 6:**
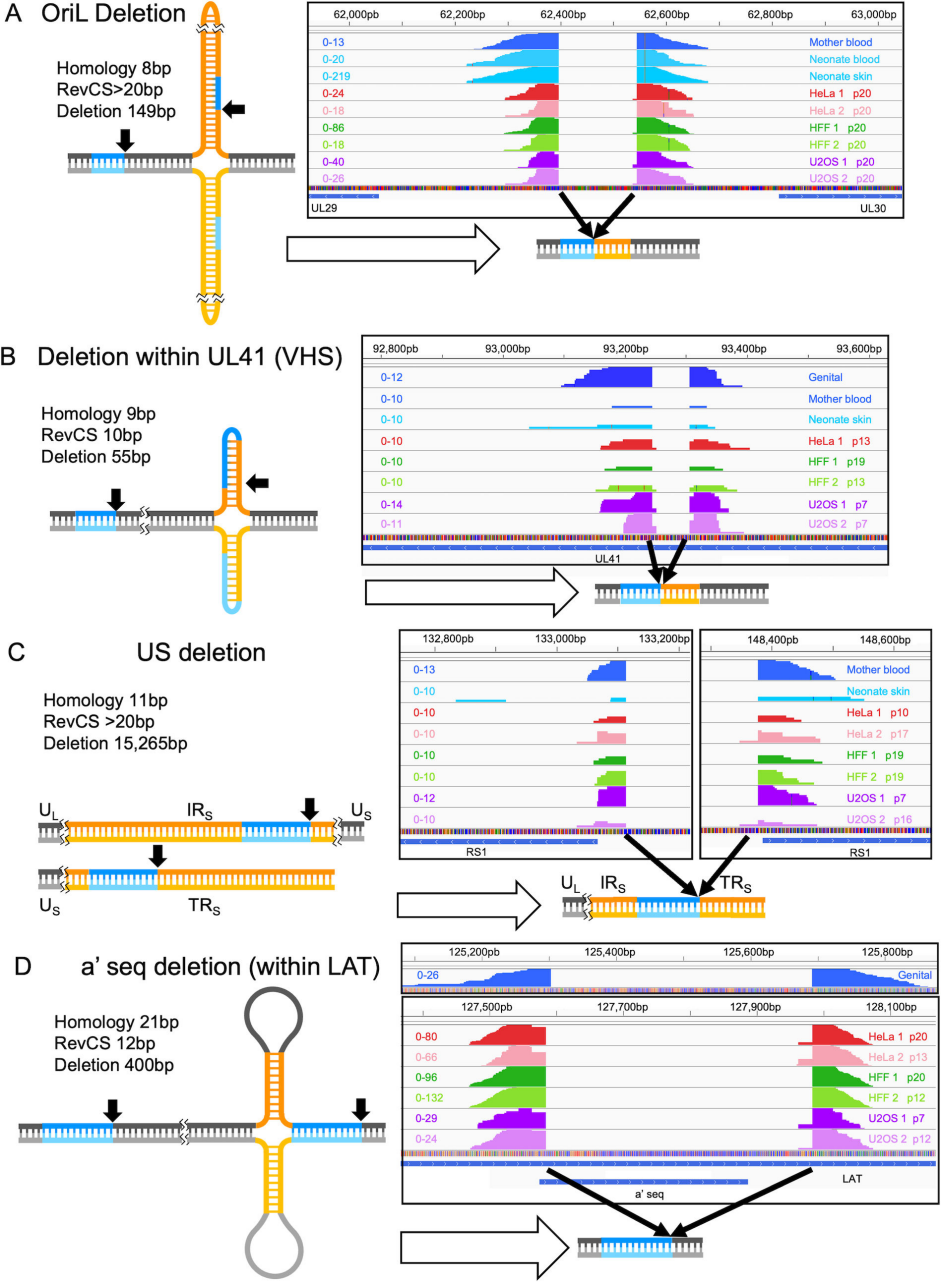
Examples of NCRs observed *in vitro* and in clinical isolates. Sequencing reads supportive of four deletions in the OriL (**A**), UL41 gene (**B**), entire US (**C**), and within a’ sequence (**D**) found in both clinical samples and in our passaging samples: HeLa, HFF, and U2OS (one passage from each cell line replicate is presented), as indicated for each sample. The coverage scale denotes for each sample. Schematic illustration of the sequence before the rearrangement event occurs in which homology sequences are marked in blue, RevCS regions are marked in orange and the rest of the sequence is marked in gray. Black arrows mark the start and end site of the NCRs and the resulting sequence is also illustrated.

Several of the NCRs identified in the clinical isolates were also detected in viral genomes from our cell line passaging. Figure 6 shows four examples of NCRs that have exact site microhomology and RevCS (one-sided or two-sided). OriL is an example of a 149-bp deletion in a previously suggested hairpin structure (Fig. 6A) (33). This same deletion was detected in the mother’s blood, the newborn blood, skin samples, and all seven cell lines (including p0). Another short deletion (55 bp) was detected in the open reading frame of UL41, virion host shutoff protein (VHS). Interestingly, the microhomology is positioned within the possible stem loop structure (Fig. 6B). This same deletion was found in the genital sample, the mother’s blood, the neonate skin, and in five out of the seven cell lines (not in HeLa1 or in p0). An example of large rearrangement that might exclude the entire US region is located between the terminal and internal repeat regions (TR and IR respectively), if positioned on the same genome (Fig. 1A). Since IRs and TRs are reverse complementary to each other, a very long RevCS was found (as expected). This same NCR was found in the mother’s blood and the neonate skin samples, in addition to all cell line replicates not including p0 (Fig. 6C). An example for one-sided RevCS is a deletion of the a’ sequence (Fig. 6D). A 400-bp deletion was found in all cell line replicates, not including p0. An identical junction was found also in the genital isolate in which only 389 bp were deleted (due to sequence changes). In the cell line replicates, next to 21 bp microhomology, a 12-bp RevCS with 28 bp apart is detected, only on one of the parental sequences however, this RevSC is not detected in the genital sample.

These examples show that NCRs detected within undiluted passaging in lab samples can also be found in the clinical samples tested, and support our finding that microhomologies and RevSC are sequence properties that are commonly associated with NCRs of HSV-1.

## DISCUSSION

Here, we used undiluted passaging in three different human-derived cell lines to explore the emergence of HSV-1 genetic diversity. We found that the amplitude and frequency of the cyclic pattern observed for DVGs differ among the cell lines, and are in accordance with the average number of viral genomes that start replication per cell in each cell line. Sequencing revealed that the population diversity of NCRs (including DVGs) is cell type dependent. Sequence properties (short homology and RevCS) that were prevalent in NCRs (formed *in vitro* by undiluted passaging) were even more prevalent in the four HSV-1 clinical isolates we tested.

Decades ago, two classes of DVGs were proposed and characterized for HSV-1 (class I containing OriS and class II containing OriL) (25, 33). Our results did not demonstrate contribution of OriL to the DVGs (Fig. S1). The higher probability for DVGs to include OriS instead of OriL might be explained by the proximity of OriS to the PAC in the HSV-1 genome, and also the presence of two OriS duplicates (Fig. 1A).

We compared two cancer cell lines (U2OS and HeLa), and immortalized fibroblasts (HFF) in our serial passaging experiment. U2OS and HeLa represent the highest and lowest, respectively, of the average number of HSV-1 genomes that start replication per cell in each cell line (Fig. 1B). These cancer cell lines have the widest known distribution of average number of viral genomes that initiate replication, based on our previous experiments (34, 35). In this study, our results support the hypothesis that the more genomes initiating replication, the greater the likelihood for formation and diversity of NCRs and DVGs. In the DVG cyclic pattern observed along the passages, we found that the amplitude and frequency are the highest and lowest, respectively, in the U2OS and HeLa cells (Fig. 1D). Furthermore, the NCRs for the two replicates of HeLa are the least diverse and U2OS are the most diverse (Fig. 3C). However, U2OS NCRs still share many common features, including clustering in the short repeat regions (Fig. 3B), distances (Fig. S3B), and the most abundant positions (Fig. S4). Similarly, NCRs found in p0 are more preserved in the HeLa passages compared to the other cell lines (Fig. 3C). Our results suggest that cells which allow more replication compartments to initiate from single genomes, such as U2OS, have an increased probability for complementation and thus accumulation of DVGs per cell (46). This may lead to rapid changes in the cyclic pattern of DVGs (Fig. 1D). We speculate that the recurrent bottleneck in the U2OS passaging results in higher diversity in the NCRs between the replicated genomes and thus reduces the possibility that few NCRs will accumulate.

We detected very few (124) minor variants above 10% in all cell lines. Most of these minor variants show a decrease in p21, indicating they may be carried on DVGs. Out of the 124 minor variants, 12 (10%) unique minor variants were found in proximity (within 10 nucleotides) of the NCRs, suggesting the possibility that these minor variants are mis-annotated. The minor variant found in gE protein (Fig. 2D) shows a slight decrease in p21, and although changing an amino acid, gE protein knock-out does not have a major effect in cell culture (47). No minor variant was accumulating consistently in all cell types, except for the upstream ICP22 change (Fig. 2B).

The NGS sequencing method requires PCR amplification of the DNA library prior to sequencing, thus resulting in a concern for artificial chimeric reads (48, 49). One of the major sources of this concern is the use of reverse transcriptase (RT) enzyme that is known to switch templates during replication (48); however in our experiments, no RT was used in the library preparation. Further, the clinical sample libraries were generated using a different preparation method [KAPA kit (44, 45)] and showed similar NCRs. Additionally, the specific patterns of NCRs were maintained during the passaging within each replicate, indicating that the observed NCRs cannot be the result of random artificial errors. Finally, the similarity of NCRs between the biological replicates (within the same cell line) and the differences observed between the cell lines further increase our confidence that the observed NCRs are biologically relevant. Thus, NGS provided a precise characterization of the NCR junctions forming DVGs. We focused, in this study, on NCRs following undiluted passaging. Although not all NCRs junctions are part of DVG populations, we see a significant decrease in passage 21 in all cell line replicates, indicating that many of the discovered NCRs are related to DVGs (Fig. 3D; Fig. S5).

**Fig 7:**
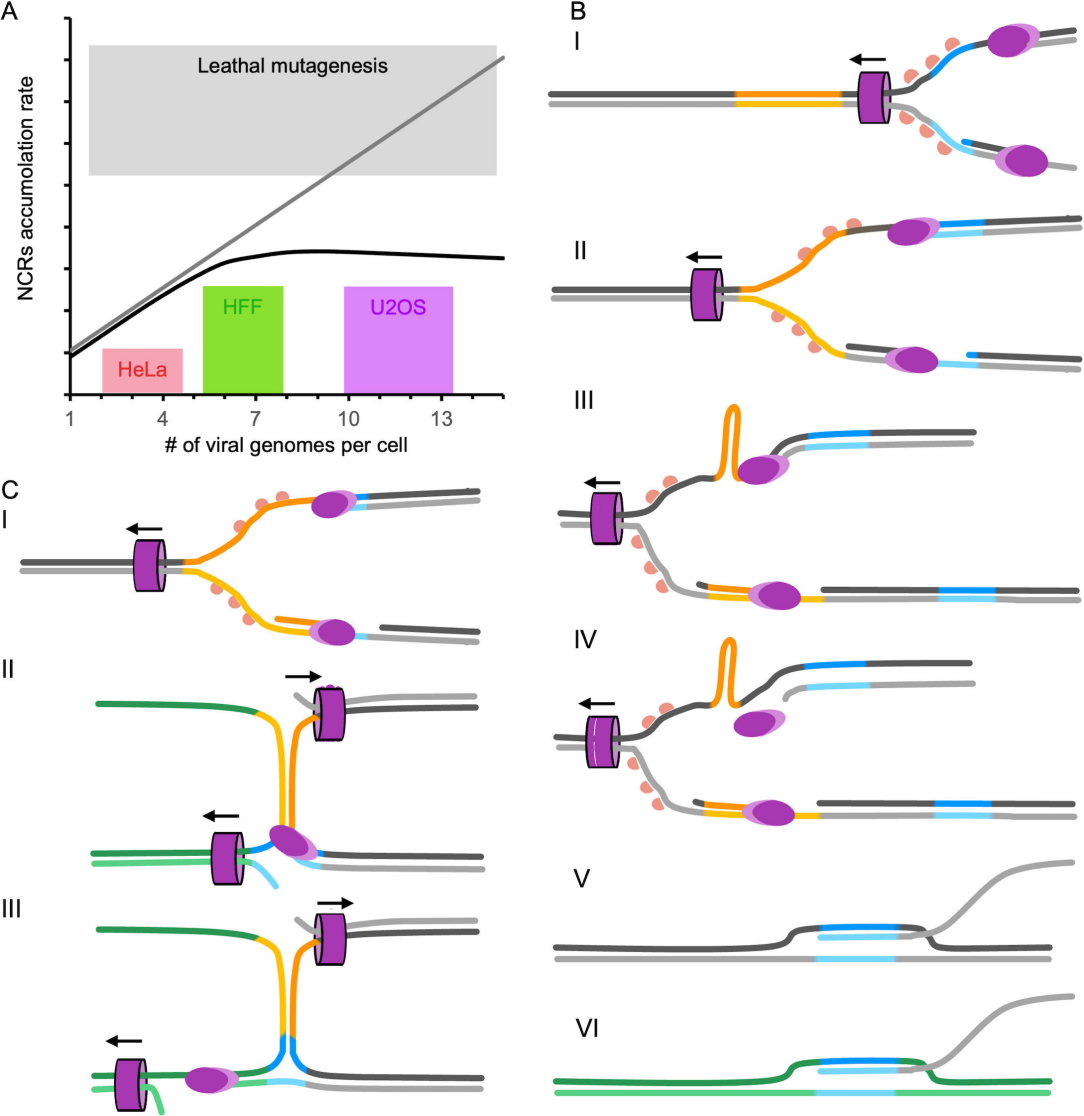
Models for NCRs formation and accumulation. (**A**) A model comparing the NCR accumulation rates to the number of genomes initiating expression in each cell. We hypothesize that the more genomes per cell will allow more complementation and thus more NCRs (gray line); however, our observed results in HeLa, HFF, and U2OS (colored bars as indicated) suggest that the NCR accumulations reach a plateau (black line). We suggest that the bottlenecks resulting from DVG accumulation can provide an explanation for this phenomenon, and prevent the virus from reaching lethal error catastrophe ranges (light gray box). (**B–C**) Models suggesting a possible pathway for NCR creation when RevCS are at one side of the parental sequences (**B**) or when RevCS are between the parental sequences (**C**). (**B I**) Viral replication fork [viral polymerase (purple oval shape)], helicase (purple barrel shape with black arrow annotating the direction of its movement), and single strain binding proteins (in shades of pink) moved on both strains of the viral genomes. (**B II**) The viral helicase moves though a region with reverse complement sequence (orange), allowing the formation of secondary structure (B III). The secondary structure prevents the viral polymerase from continuing the replication process (**B IV**). This allows the newly synthesized DNA to hybridize with micro-homologous sequence (blue) to other places in the same genome (**B V**) or in a nearby genome (**B VI**), creating a new junction site NCR. The alternative model (**C**) color coded as (**B**) suggests that region with reverse complement sequence (orange) once becoming single stranded due to the movement of the replication fork (**C I**) can interact with the complementary sequence on adjacent replication fork (**C II**). The newly formed secondary structure provides the opportunity for the polymerase to switch template [based on sequence homology in blue (C III)].

Surprisingly, although the number of initiating genomes in U2OS is twice that of HFF, the number of new NCRs forming in the U2OS replicates over all the passages is not significantly different from HFF replicates (Fig. 3D). One possible explanation for these discrepancies is that higher frequency and amplitude of the DVGs (such as in U2OS) lead to population bottlenecks which decrease the complementarity and limits in the number and types of the DVGs (Fig. 7A). Our model suggests that the bottlenecks resulting from DVG accumulation have a role in maintaining population diversity that prevents viral population extinction by lethal mutagenesis (due to rearrangement).

Aligning the total different NCR population along the HSV-1 genome showed cell line-specific patterns (Fig. 3B; Fig. S3B). Similarly, a unique pattern for the most prevalent NCRs for each cell type was observed (Fig. S4). This cell type preference was maintained between replicates and over all passages of the study (Fig. S4 and S5). In the HFF replicates, NCRs junctions are present throughout all the HSV-1 genome, while in the U2OS replicates, NCRs are limited to the US region (Fig. 3B). Although most of the NCRs in the p0 sample remain in all cell line replicates (Fig. 3C), infections of HeLa cells do not accumulate many new DVGs and they show a similar pattern to p0 (Fig. S4 compared to 2B and Fig. S3). These results together suggest that cell type intrinsic properties influence the most prevalent NCR junction positions used along the genome.

Microhomology DNA repair is a known mechanism of eukaryotic cells that has been studied intensively due to the relation to oncogenesis. Microhomology is defined by 2 bp–20 bp homology, and the homology has mostly been reported on exact junction sites, although in some cancer cell rearrangements, the homology is positioned few base pairs from the junction site (42, 43, 50). Our analysis identified significant increase over random sequences only from 7 bp homology. Fig. 4B and 5A suggest that, in some cases, longer than 20 bp homologies are found; indeed we observed in some cases longer homologies by eye. Overall, 82% and 30% of NCRs were found with short homologies (7 bp–20 bp) and only 62% and 37% on the exact junction site in clinical isolates and in our undiluted passages, respectively. These discrepancies (the longer homologies and the lower prevalence of exact junction site) might suggest that NCRs with short homology that we have observed are not necessarily mediated through the classical microhomology DNA repair pathway. On the other hand, it was suggested that longer microhomology results in more frequent repair (51), which is similar to the prevalence of longer homologies NCRs in our data (Fig. S6A).

In our NCRs population, we see a deletion in OriL among all cell lines (including p0) and three out of the four clinical isolates examined here (Fig. 6A). OriL deletion in HSV-1 has been reported previously in bacterial artificial chromosome (BAC) containing the viral genome and other HSV-1 isolates (33, 52). OriL deletion is not decreased in p21 (Fig. S7A), corroborating previous findings that OriL-deleted viruses still produce replicating genomes (52). We speculate that the high degree of secondary structure in OriL may result in a hot spot for NCR junctions. However, VHS out of frame, US, and the a’ sequence deletions show oscillations in frequency throughout the passaging and a significant decrease in p21, suggesting these are DVGs (Fig. S7B through D).

Interestingly, RevCS (i.e., possible secondary structure) are observed in 72% of the clinical isolates and 30% in our undiluted passaged virus genomes. This supports the role of RevCS as hot spots for NCR junctions. We suggest a model for viral NCR formation for either RevCS on one parental sequence (Fig. 7B) or for RevCS between the two parental sequences (Fig. 7C). During DNA annealing in the replication process, RevCS may result in temporary secondary structures in the case of one parental side (Fig. 7B I–III). If formed, these secondary structures may sterically interfere with the viral polymerase progress (Fig. 7B III). The stalled polymerase may release the newly synthesized DNA (Fig. 7B IV) which will hybridize with micro-homologous sequences found either on the same genome or on an adjacent genome (Fig. 7B V and VI). Alternatively, RevCS between two parental sequences were found with longer RevCS (Fig. 4G). These longer RevCS may induce secondary structures between adjacent replication forks (Fig. 7C I–III). These structures may provide the opportunity for polymerase template switching, especially in the presence of homology sequence (higher frequencies of homologies and exact homologies are found in the two-sided Fig. 4F). These models explain the most frequent NCRs we observed (especially in the clinical samples) and fit with the isomerization and formation of replication branching intermediates previously observed during HSV-1 infection (53).

We hypothesize that our *in vitro* conditions may have induced more NCRs than commonly seen during natural infection, partially due to the observation that some of our NCRs start to accumulate after passage 12 (Fig. 3D). This can explain the higher number of different NCRs and the higher percentage of NCRs that do not have short homology and RevCS in our samples compared to the clinical isolates. Therefore, we speculate that our suggested models (Fig. 7B and C) represent most NCRs that occur during natural infection; however, there are probably additional mechanisms for the creation of NCRs since not all NCRs have these properties.

Here, we suggest a possible beneficial outcome of HSV-1 DVGs that prevents lethal accumulation of genetic rearrangements. This might be a common feature of DVGs in other viruses as well. Furthermore, we identified frequent sequence properties (short homology and RevCS) within the HSV-1 NCRs. We suggest that these properties drive the NCR junction sites and might be common to other DNA viruses.

## MATERIALS AND METHODS

### Cells

The experiments are performed with Vero (African green monkey kidney epithelial cells, ATCC CCL-81), HFF, U2OS (human female osteosarcoma cells, ATCC HTB-96), and HeLa (human female cervical cancer, kind gift from Eran Bachrach labratory) cells. Immortalized HFF by human telomerase reverse transcriptase (hTERT) transfection were a kind gift from the Sara Selig laboratory. All cells are grown with Dulbecco’s Modified Eagle Medium (DMEM X1; Gibco), supplemented with 10% fetal bovine serum (FBS; Gibco) at 37°C with 5% CO2. For virus plaque assays, a semi-solidified DMEM was used containing (hydroxypropyl) methylcellulose 5 µg/mL (Sigma), 2% FBS, 2% NaCHO₃, and 1% PenStrep.

### Three-color assay

To estimate the number of HSV-1 genomes expressed in each infected cell, either U2OS, HFF, or HeLa cells were infected with an even mixture of three isogenic florescent viruses, OK11, OK12, and OK22 (35), each expressing a unique fluorescent protein under the immediate early cytomegalovirus (CMV) promoter: mCherry, EYFP, and mTurq2, respectively, at an MOI of 20. At 6–8 hours post infection (HPI), the infected cells were imaged using a Nikon Eclipse Ti-E epifluorescence inverted microscope. The experiment was repeated three times. From an individual well, five random areas were imaged. Three images from each well were analyzed for the presence of the three colors, with at least 200 cells analyzed in each. To estimate the most likely average number of genomes expressed in each cell (λ) according to the number of one - (*r*1), two - (*r*2), or three-color (*r*3) cells out of the number of cells analyzed (*n*), we used the model we have previously developed (22):


λ=−3ln(1−(r1+2r2+3r3)/3n)

### Serial undiluted passages

A stock of viral recombinant OK22 was prepared on Vero cells from a single plaque. One plaque was collected under inverted fluorescent microscope and was propagated for 72 HPI on a single 10 cm plate pre-seeded with Vero cells (to form the primary stock). A working stock was collected following 48 HPI for another plate covered with Vero cells with 200 µL of primary stock. For the first passage, 1 mL of the working stock was placed on 10 cm plates pre-seeded with either HFF, U2OS, or HeLa cells at MOI 20. Two replicates for each cell lines were infected. At 1 HPI, the cells were covered with an additional 9 mL of medium and further incubated. At 48 HPI, 50 mM HEPES buffer was added and cells were scraped into the medium and collected. An aliquot of 2 mL from the 10 mL collected viral lysate was used to infect the next passage and the rest were kept for further analysis (viral titer, qPCR, and viral DNA purification). This process was repeated for 20 passages. The last passage (p21) was done after the last stock was titered, and infection was carried out at MOI 0.01 and collected after full cytopathic effects (CPE).

### Quantitative PCR

#### Sample preparation

To prepare DNA samples for analysis, each experimental sample from the 20 passages was incubated with lysis buffer 10 mM Tris-HCl, pH = 8.0, 1 mM EDTA (Merck), 1% Tween 20 (Sigma-Aldrich), and 0.04% proteinase K (BIO-LAB, Israel). We incubated for an hour at 37°C and heated for 10 minutes in 95°C to disassemble the proteinase K. Cell lysates were directly used for PCR template. The primers are listed in the Table S1.

#### Analysis

qPCR was carried out with QuantStudio 12K Flex (Applied Biosystems) using SYBR master mix (Applied Biosystems). OriS, OriL, and PAC cycles were compared to control UL11 (previously used in our lab as control for viral genes) within the sample, and to purified viral DNA as a control between samples.

### DNA isolation

For each cell line replicate at each passage, 6 mL of viral lysate was centrifuged in 1,500 relative centrifuge force (RCF) for 5 minutes at 4°C. The medium after the centrifugation was stored. The pellet was washed once with phosphate-buffered saline (PBS) buffer and centrifuged another time, and then the buffer was aspirated. After two freeze/thaw cycles at −80°C and 37°C, the pellet was suspended in Tris magnesium (TM) buffer (10 mM Tris HCl, pH 7.5, 30 mM magnesium chloride). The cells pellets were sonicated for six pulses each 15 seconds, and cells debris were removed by 5 minutes of centrifugation at 9,000 RCF. The stored media were centrifuged for 1 hour in 100,000 RCF at 4°C. The medium pellet was merged with the cell supernatant and the samples were incubated in 2mL of Benzonase (250 units/mL) and RNase (40 µg/mL) for 1hour at 37°C. To purify the viral capsids, we used 30% sucrose cushion below the samples and centrifuged for 1 hour in 100,000 RCF at 4°C. The supernatant was removed, and the capsid pellet was washed once with Tris NaCl and EDTA (TNE) buffer (10 mM Tris-HCl pH 7.5, 150mM NaCl, 1mM EDTA) and centrifuged for 10 minutes in 100,000 RCF at 4°C. The capsids were resuspended in TNE supplemented with proteinase K 100 mg/mL, 0.5% SDS, and 20 mM EDTA, and incubated for 2 hours in 50°C. Phenol-chloroform was added and the emulsion was centrifuged for 5 minutes at 8,500RCF at room temperature. This was repeated until no proteins were visible. DNA was purified with ethanol participation, resuspended in Tris EDTA (TE) buffer (10 mM Tris-HCl pH 7.5, 1 mM EDTA) and stored in −80°C.

### Illumina NextSeq library preparation

Illumina NextSeq library preparation was performed using NexteraXT 250 bp, paired-end reads protocol described in reference (54). Briefly, three stages were included: tagmentation using Amplicon Tagment Mix enzyme and TD (Tagment DNA) buffer, PCR using superphi enzyme to insert indexes, and size selection using Ampure beads. We also tested in two samples addition of GC enhancer in the PCR section to optimize DNA polymerase complex HSV-1 GC rich template. In the samples with the enhancer, the number of total reads was lower; therefore, we did not continue to use the enhancer for the rest of the library preparation (see Table S2). The DNA content was tested by Qbit and the distribution of read length using Agilent TapeStation.

### Illumina NextSeq deep sequencing, processing, and quality assurance

#### Sequencing

Libraries were pooled and sequenced in six batches (see Table S2). Preparation of libraries for loading on NextSeq 550 system, 300 cycles, paired-end, were prepared according to Illumina protocol including preparation of denatured PhiX library according to Illumina instructions as a control for the sequencing (see Table S2 for running statistics)

#### Processing and quality assurance

Fastq files, generated from NextSeq runs, were concatenated for each sample from the four different lanes. FastQC tool (55) was run for QA. We used cutadapt (56) for adapters removal and quality trimming using quality cutoff parameter of 20 and minimum length of read of 20 bp. Paired-end adapters that were used were AGATCGGAAGAGCACACGTCTGAACTCCAGTCAC and GATCGTCGGACTGTAGAACTCTGAACCTGTCG.

### p0 assembly

We assembled p0 reads using Spades 3.13 (57) paired-end assembly with --careful --cov-cutoff off -t 18 m 100 parameters. We blasted the output contigs to the OK22 (HSV-1 strain 17 with a fluorescent protein gene addition between UL37 and UL38) genome and filled the missing areas with OK22 genome.

### Alignment

Alignment for human (Genome Reference Consortium Human Build 38) was run with paired-end reads using Bowtie2 (58) and default parameters. Human unaligned reads were extracted using samtools (59) and aligned using Bowtie2 to the p0 assembled genome.

### Comparing qPCR analysis and sequencing OriS accumulation over the passages and cell lines

For each sample and cell line replicate, human unaligned reads were BLAST for OriS and UL11 coding region, sequences with the following parameters: -dust no -soft_masking F -perc_identity 85 -evalue 1e-10. The number of OriS aligned reads was divided by UL11 aligned reads for each passage and cell line replicate.

### AccuNGS

HSV-1 genome is composed of repeats and is longer than the RNA genomes that were used in AccuNGS development (39). Thus, we needed to do several adjustments for HSV-1 genome. First, we aligned all the human unaligned reads to the assembled p0 without TRs using BLAST 2.7.1. Using in-house script (Filter_blastn_outputs.R), we extracted only two best 2 e-value alignments for each read from BLAST results. Since each paired-end read was represented as one read, the two alignments represent alignments for both ends.

The frequencies and variant calling of AccuNGS were run on those filtered BLAST outputs, and we detected all minor variants that were prevalent more than 10%. For all the substitutions and positions that were above 10%, we extracted its frequencies from all samples (all passages and cell lines).

### ViReMa

Human and viral unaligned reads from all samples were run separately as ViReMa (41) input. p0 assembled genome was used as reference. To simplify the analysis, we initially used a genome sequence containing only one long and short repeat by eliminating the TRs at the ends of the genome. When running the alignment for ViReMa with the full sequence, we noticed that we are missing recombination events that occur from the terminal repeat to the internal repeat (or vice versa). We added these repeat regions NCRs to our analysis.

The parameters for the run were --Defuzz 5 (to report at 5´ end of fuzzy region) --N 1 (number of mismatches tolerated in mapped seed and in mapped segments) --X 8 (number of nucleotides not allowed to have mismatches at 3´ end and 5´ of segment) --Pad 250 (are required if recombination occurs as end of genome).

### ViReMa post processing: adjustments for long and repetitive HSV-1 and reproducibility

#### Adjustments for long and repetitive HSV-1

For each sample and passage, we extracted the NCRs represented by their start and end positions along with supporting reads. We filtered only NCRs whose supporting reads indicate matches to two different positions in the HSV-1 genome. Specifically, these reads included the sam file pattern: “match-gap-match,” where match and gap >10 bp. Therefore, alignments with edge trimming only were discarded from the analysis. In addition, we discarded from the analysis any read whose “match-gap-match” pattern was inconsistent with the NCRs start and end positions they supported.

#### Creating a complete NCRs table for all passages and cell line replicates

We calculated for each NCR its normalized reads by the following equation: number of supporting reads / (total reads aligned * 1,000,000). Samples that were sequenced in several batches were summed for their normalized reads for further analysis. NCRs with less than 10 supporting reads in all *in vitro* samples (per passage per cell line) were discarded from further analysis (Table S3).

#### Reproducibility

Eight samples with low coverage were repeated for library preparation and sequenced in a different batch. For each DNA source, the correlation between the two sequencing runs, pre- and after ViReMa post processing, was calculated (Table S4), indicating that the NCRs detected were reproducible.

#### Clustering NCRs

An in-house script (Cluster_NCRs_by_range.R) was used to cluster adjacent NCRs start and end positions as we assumed minor deviations are likely to be analysis imperfection rather than real new NCRs. We clustered a range of 1–20 nucleotides distance in the NCRs start and end positions (see Fig. S8). The number of NCRs decreased substantially (a decrease of about 20% in the number of NCRs for the first four nucleotides and only 2.5% decrease between nucleotides 5 and 10). Therefore, all NCRs that were up to five nucleotides apart in the start or end position were clustered together and summed up for their normalized reads yielding 806 unique NCRs over all the cell types replicates.

### Coverage plots

ViReMa detects all rearrangements including deletions, insertions, and inversions. These insertions are denoted by alignment end position that are lower than the start position. In HSV-1, there are four isomers that invert either the UL and US regions (Fig. 1A); thus, it is not possible to distinguish between HSV-1 isomers and inversions [as most NCRs detected had at least one position in repeat region (Fig. 3B)]. Thus, we treated all the NCRs as deletions in these plots. We summed the frequency of its supporting reads, for each cell line replicate, at all positions between the start and end of each NCR event. These total normalized reads of each genomic position for all NCR events spanning over this position were summed either for all passages together or per passage.

### Homology detection

An in-house script (Find_microhomology.R) was developed to detect the longest homologous sequence within 7 bp–20 bp, between the NCR start and the end parental sequences (Fig. 4A). The script reported the genome positions of the beginning of the homology sequences detected. Homologies (no mismatches allowed) of length 20 to length 7 were searched between the start +/−20 bp sequence and the end +/−20 bp sequence. When the distance between the end and start positions was shorter than 40 bp, the search was done on the entire sequence (−20 bp from start to +20 bp from end). If homology was found, it was reported (i.e., other homologies in the same length or lower length were not searched). We randomly selected 2,000 positions for the start and end positions. The same in-house script was used for the homologous sequence detection. Length that was 7 bp or more showed deviation from random, thus presented in the results.

### RevCS detection

An in-house script (Find_RevCS.R) was used to detect the longest reverse complimentary sequence between the NCR start and the end parental sequences (Fig. 4A). The assumption was that short secondary structures can be detected on either side of the parental genomes or between them. For each parental genome junction site, 50 bp were added to the direction of the other parental junction position and 25 bp to the opposite direction. If the width of the NCR junction was smaller than 100, the NCR was added to the one-sided RevCS. RevCS were searched for complementarity of 14 bp to 9 bp. RevCS of longer than 14 bp accumulated in the 14bp RevCS. We randomly selected 2,000 positions for the start and end positions. The same in-house script was used for the RevCS detection.

### Clinical samples

NGS data for four uncultured clinical samples were obtained from an HSV-1-infected mother (blood) and her neonate (blood and skin samples) (44) and one unrelated genital sample that was previously shown to have a high frequency of minor variants (45). We ran ViReMa on unaligned sequence reads from each sample and used its *de novo* assembled consensus genome as reference genome. We also run ViReMa with the assembled p0 genome as a reference to compare the NCRs positions to the cell line positions.

## Data Availability

All raw and processed data were uploaded to SRA at the following link: http://www.ncbi.nlm.nih.gov/bioproject/955802. All in-house scripts are available at https://figshare.com/s/ce9cb142c57fe5047575.

## SUPPLEMENTAL MATERIAL

Supplemental file jvi.00955-23-s0001.pdf Eight supplemental figures and Table S1.

Table S2 jvi.00955-23-s0002.xlsx Sequencing info.

Table S3 jvi.00955-23-s0003.xlsx Comparison of NCRs from different NGS libraries and sequencing batches of the same samples.

Table S4 jvi.00955-23-s0004.xlsx Total NCRs detected from viral undiluted passaging experiments.

ASM does not own the copyrights to Supplemental Material that may be linked to, or accessed through, an article. The authors have granted ASM a non-exclusive, world-wide license to publish the Supplemental Material files. Please contact the corresponding author directly for reuse.
