# Supplementary material for: Shared sequence characteristics identified in non-canonical rearrangements of HSV-1 genomes
Source: J Virol. 2023 Nov 22;97(12):e00955-23. doi: 10.1128/jvi.00955-23 (PMC10734421; doi:10.1128/jvi.00955-23)
Supplement: Supplemental file — Eight supplemental figures and Table S1. [file jvi.00955-23-s0001.pdf]

Shared Sequence Characteristics Identified in Non-Canonical Rearrangements of HSV-1 Genomes

Alina Shitrit, Valerya Nisnevich, Nofar Rozenshtein, Hila Kobo, Hoang Van Phan, Savaş Tay, Moriah Szpara, Matthew D. Weitzman, Nir Drayman, and Oren Kobiler

Supporting information

Supplementary Figure 1. OriL does not fluctuate along the passages as opposed to OriS and PAC. qPCR results for Packaging signal (PAC) (left), OriS (middle) and OriL (right) copy numbers compared to UL11 gene at the different passages are shown for each replicate in cell line. HeLa (shades of red), HFF (Shades of green) U2OS (shades of purple), darker color represent replicate 1 and the brighter for replicate 2.

Supplementary Figure 2. Correlation between sequencing and PCR results for OriS. **(A)** OriS and UL11 sequences were searched and counted using BLAST in all passages and in each cell line replicate human unaligned reads (dotted line) were compared to the OriS qPCR results (dashed lines). **(B)** Correlation between the aligned read to OriS and the qPCR results (as in a). each point represent data from one passage for each cell line replicate. R-squared is presented. Color coded as **Supplementary Figure 1**.

Supplementary Figure 3. Total number and distances of total different NCRs observed at each cell line replicates. (A) Total number of different NCRs from all passages in each cell line replicates and in p0 are shown as indicated. **(B)** The distribution of distances between the NCRs start and end positions according to the canonical isomer (P) were plotted. Each bar represents the percentage of NCRs (out of total) in the range size between the current and the previous bp distance (on the x axis). Color coded as **Supplementary Figure 2**.

Supplementary Figure 4. Coverage plots. Sum of all NCRs supporting reads (normalized) in p0 and each cell line replicates (as indicated) for the four possible HSV-1 isomers. NCRs were plotted on the assembled p0 genome (x axes).

Supplementary Figure 5. NCRs pattern throughout the viral sequence doesn't vary along the different passages. Coverage plots of NCRs junctions in isomer P changing along the passages for each cell line replicate: **(A)** HeLa1, **(B)** HeLa2, **(C)** HFF1, **(D)** HFF2, **(E)** U2OS1 and **(F-G)** U2OS2. For **(A-F)** panels the Y axis identical, and in panel **G** the Y axis is shorter so the differences could be detected. Each passage is colored as indicated in the figure legend, p0 in cyan and p21 in black.

Supplementary Figure 6. Homology and RevCS are more prevalent in NCRs that are common to many cell line replicates. **(A)** Average homology length in bp compared to the number of cell line replicates in which these NCRs appear. **(B)** Percent of exact site homology out of total homology according to the number of cell line replicates in which these NCRs appear. **(C)** Percent of RevCS above 12 bp distribution out of total RevCS according to the number of cell line replicates in which these NCRs appear. R-squared for each correlation is presented.

Supplementary Figure 7. Specific NCRs examples (shown in Figure 5) distribution along the passages and cell lines. The normalized read supporting each NCRs is plotted along the passages for each cell line replicates. OriL deletion**(A)**, Deletion within UL41 (VHS) **(B),** U_S deletion **(C)** and a' seq deletion **(D)**. Color coded as **Supplementary Figure 1**, p0 in black.

Supplementary Figure 8. Clustering of NCRs generated by ViReMa. Number of unique NCRs remaining after clustering when choosing different distance cutoffs (1-20bp) for clustering together adjacent NCRs.

Supplementary Table 1 – Primers for qPCR. Primers used in this study are listed.

Supplementary Table 2 – Sequencing Info. The table columns describe the passage, sample and batch in column one. Samples that their library preparation included enhancer are noted by "-we" suffix to the sample name the total reads in column 2, the percent of virus aligned reads in column 3, Percent of human aligned reads in column 4, the number of viral aligned reads in column 5, and percent and total number of unaligned reads in column 6 and 7, respectively. Several samples were run more than once (for several samples also the library preparation was repeated) to increase the number of reads for a low coverage samples.

Supp Table 3 – Total NCRs detected from viral undiluted passaging experiments. The table columns describe the sample name in column A, NCR start and end positions according to the p0 assembled genome in columns B and C, respectively. The number of actual and normalized supporting reads for each NCR junction in columns D and E, respectively. The number of reads aligned to the canonical parental sequence (not supporting the NCRs) at the start and end positions

in columns F and G, respectively. The presence and the length of microhomology and RevCS presented in columns H and I, respectively.

Supp Table 4 – Comparison of NCRs from different NGS libraries and sequencing batches of the same samples. This table shows for each prepared and sequenced library that was replicated the number of NCR supporting reads before and after ViReMa post processing. Before processing (columns B to D) R correlation was calculated for each sample by comparing the number of reads supporting all above 20 reads per NCRs detected in the higher coverage sample. All detected NCRs in the lower coverage sample were included in this comparison. Post processing (columns E to J) we compared the frequency of all unique NCRs in both libraries 1 and 2 (lib 1low coverage and lib 2 high coverage samples) to the other library and present the R correlation results in columns I and J respectively.

Supplementary Table 1.

Target Gene	Forward sequence	Reverse sequence
OriS	AGCTTCCTTGTTTGGAGACC	TGTCGCGCTGAGATGAATC
OriL	GAAAAGACGTTCACCAAGCTG	CTGGAGGTGCGGTTGATAAA
UL11	GACAGACGAGAATATCCAGGG	TTATCTTCCACGAACCCAGC
PAC	CCGCCGCCGCTTTAAAGG	GGCCAGACCCCGAAAACG

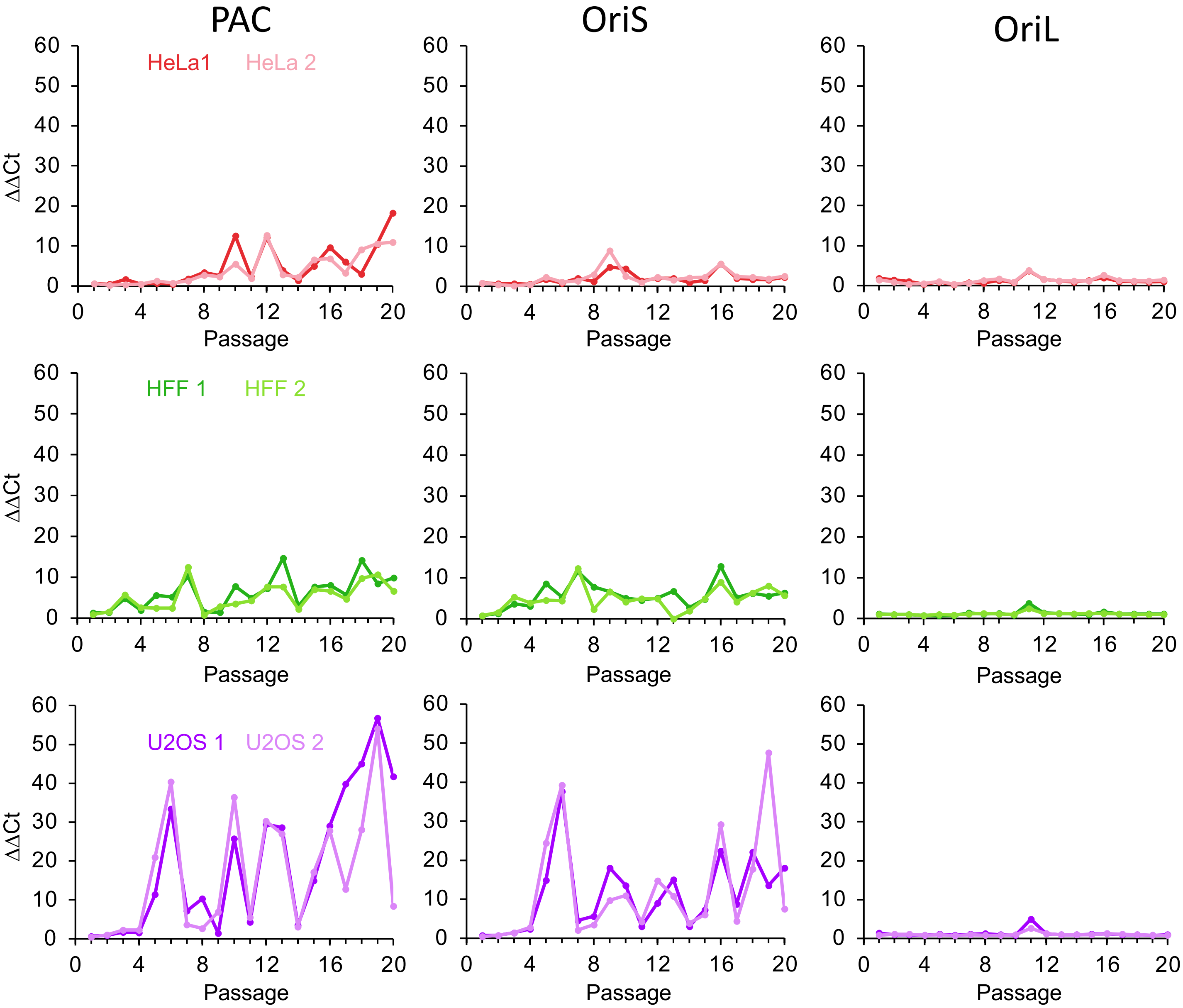

Supp. Figure 1

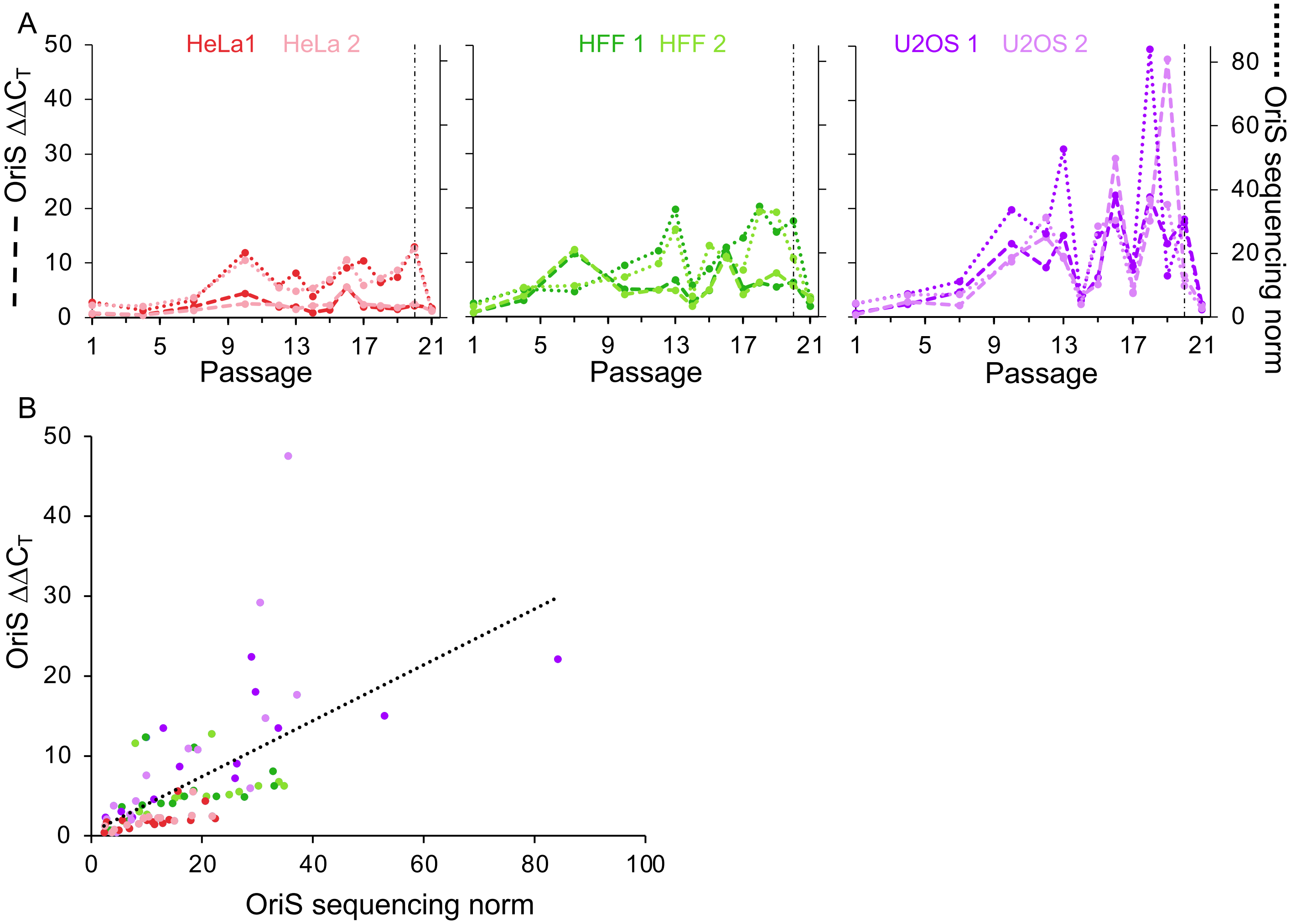

Supp. Figure 2

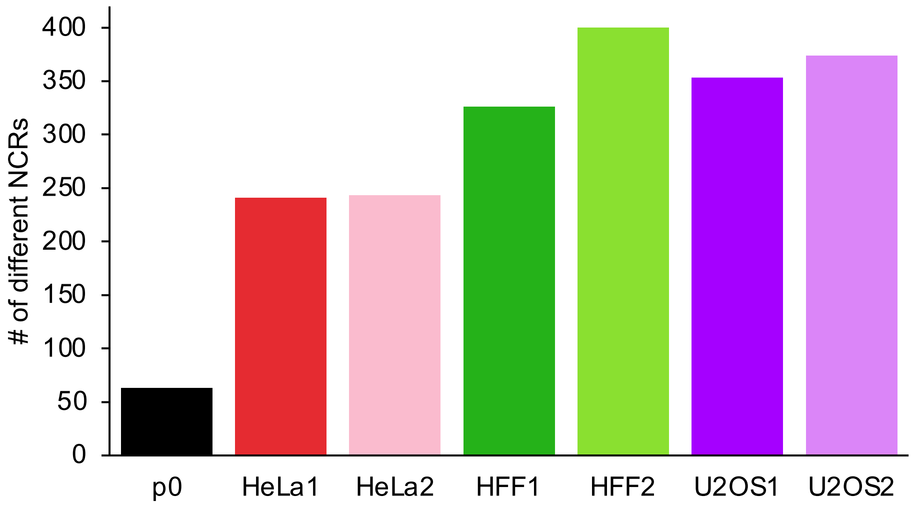

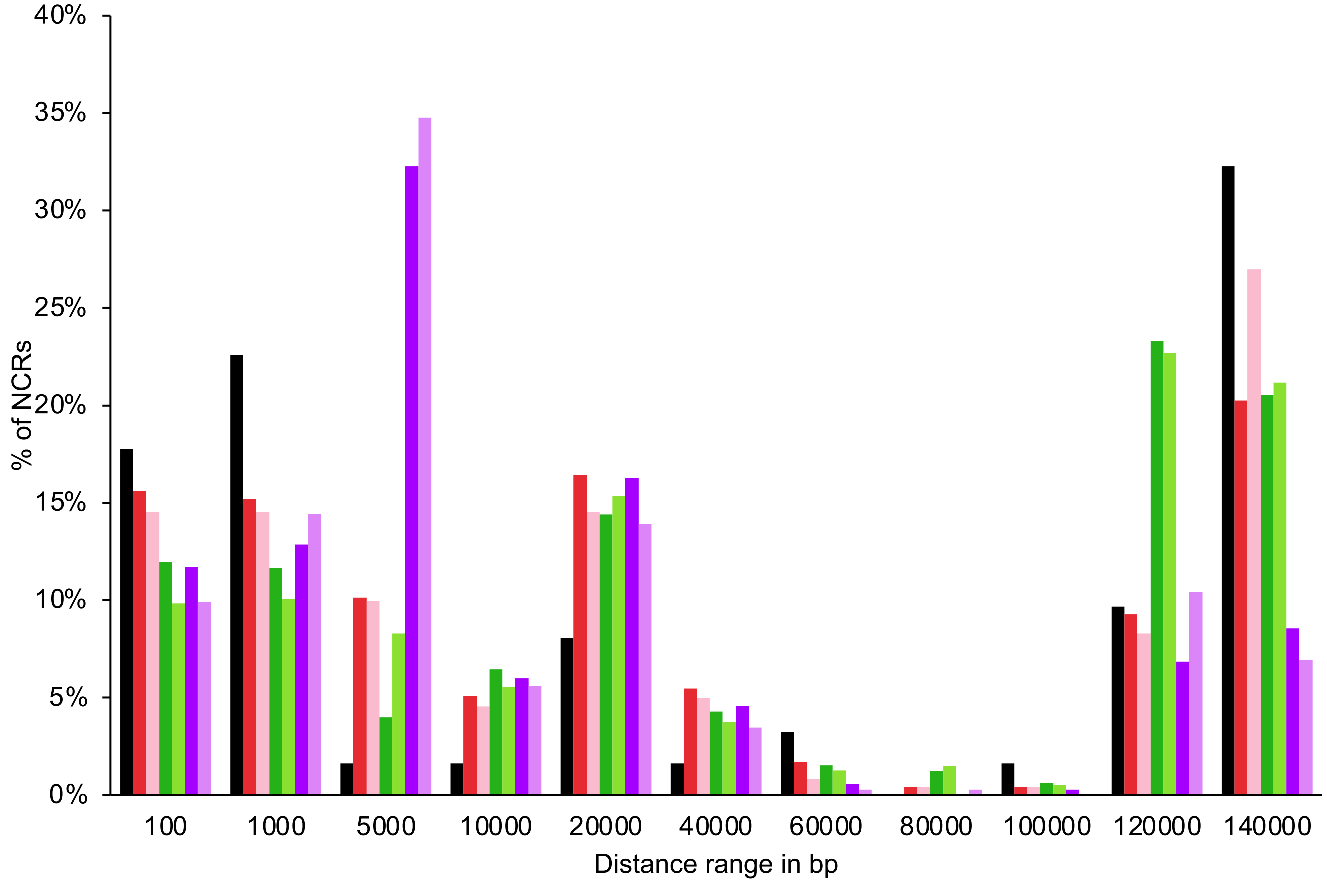

Supp. Figure 3

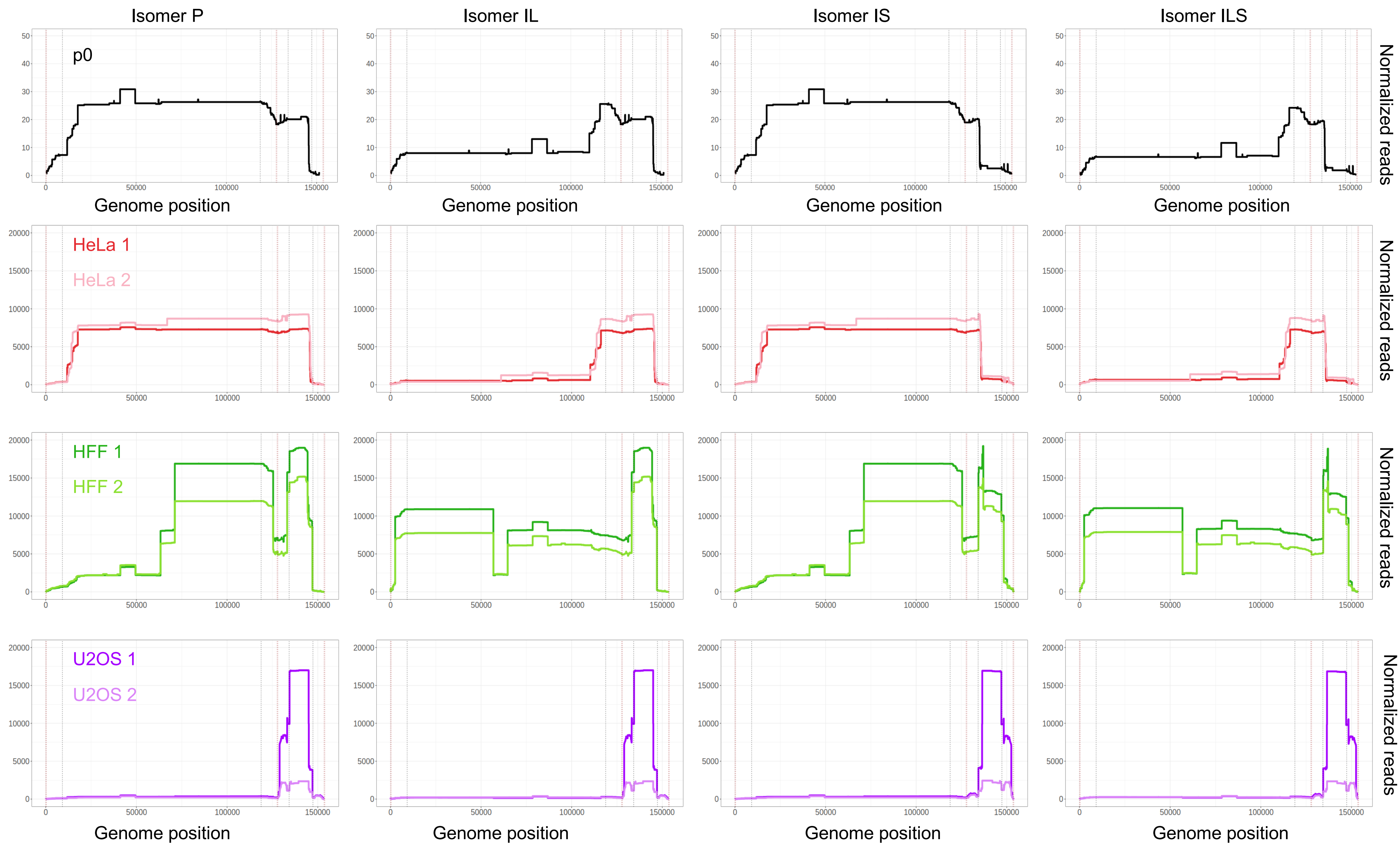

Supp. Figure 4

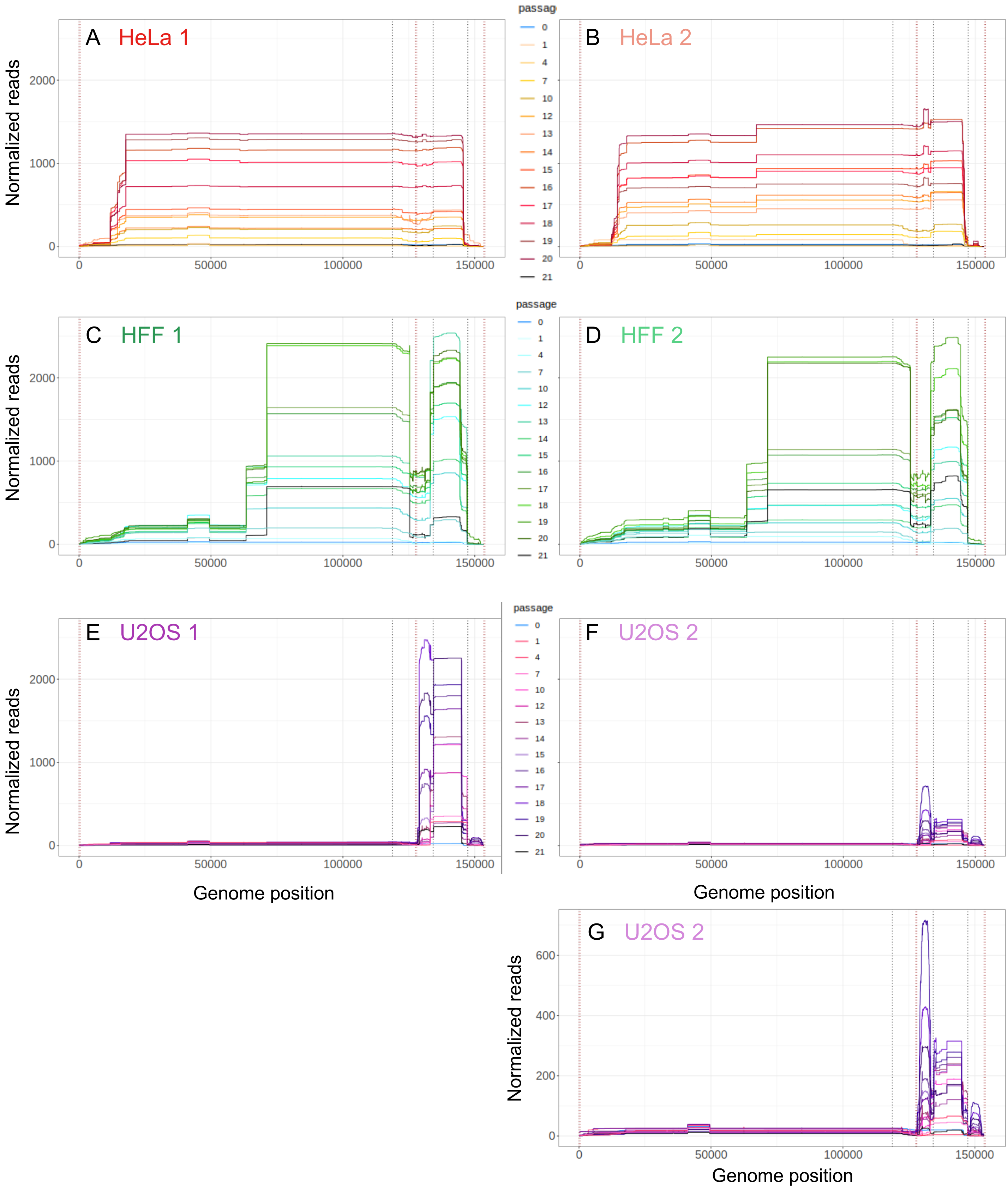

Supp. Figure 5

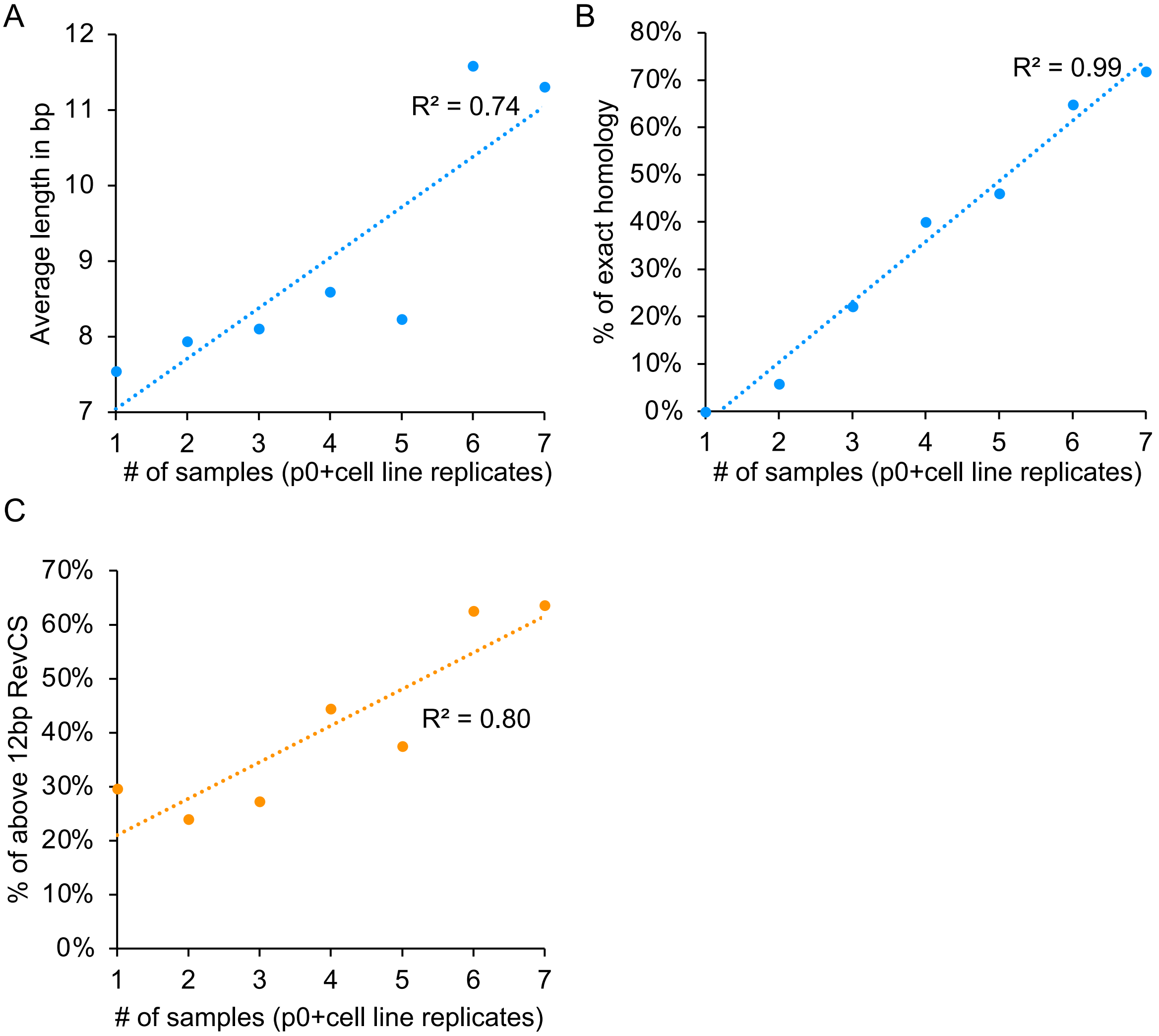

Supp. Figure 6

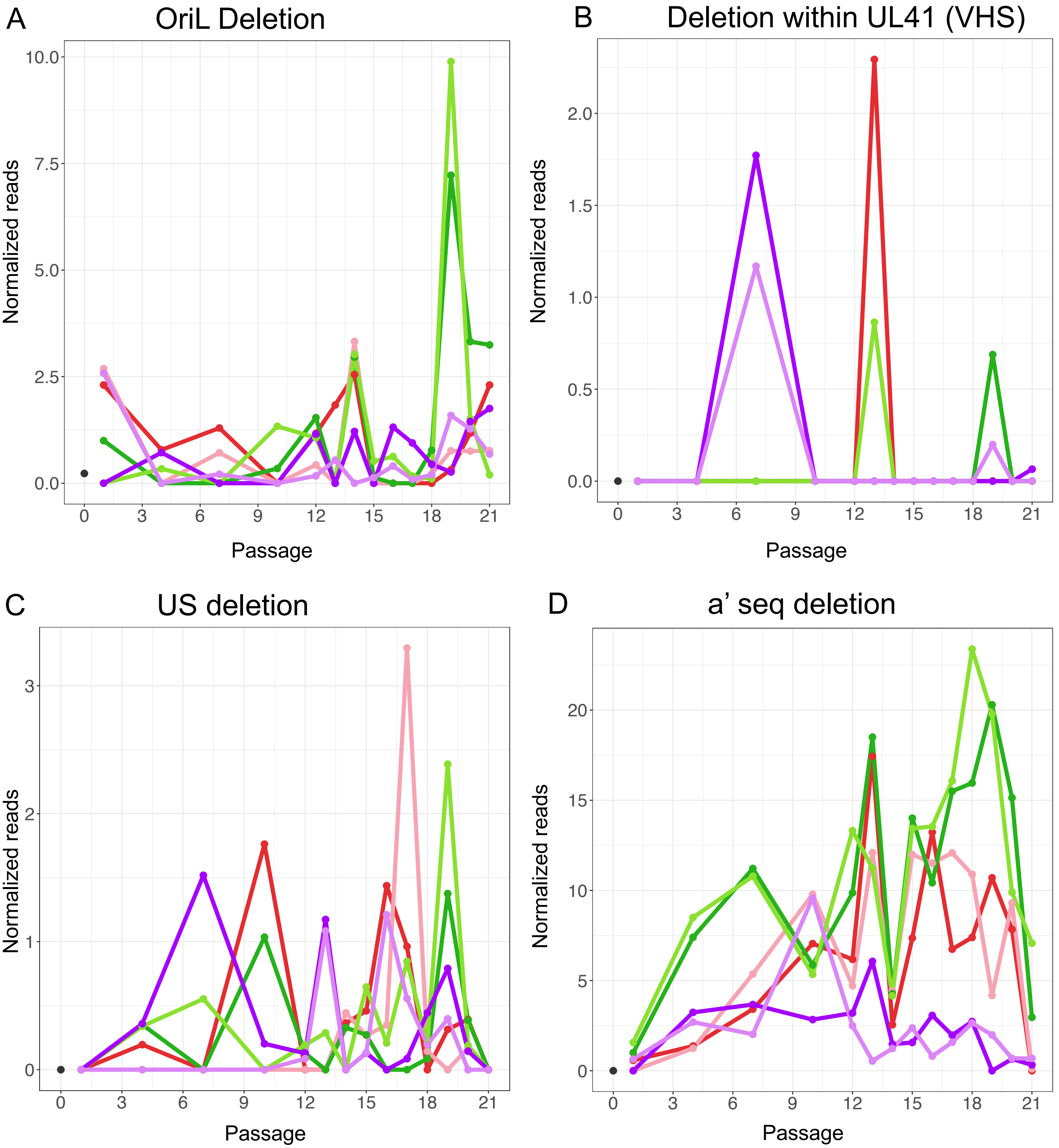

Supp. Figure 7

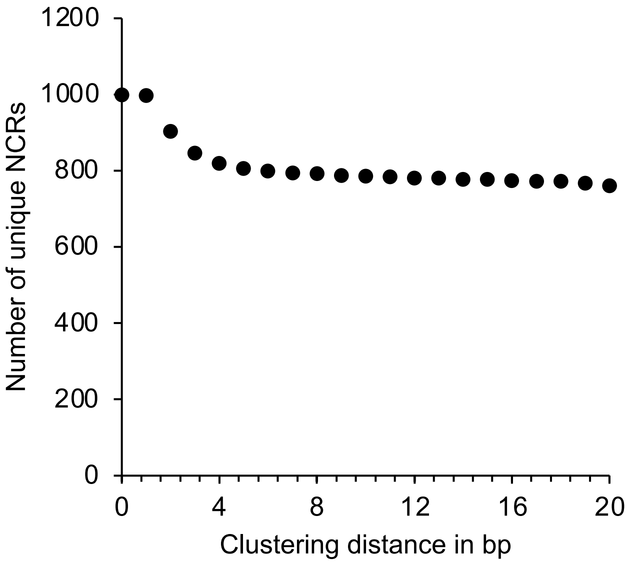

Supp. Figure 8
